# NAD^+^ precursors prolong survival and improve cardiac phenotypes in a mouse model of Friedreich’s Ataxia

**DOI:** 10.1172/jci.insight.177152

**Published:** 2024-07-18

**Authors:** Caroline E. Perry, Sarah M. Halawani, Sarmistha Mukherjee, Lucie V. Ngaba, Melissa Lieu, Won Dong Lee, James G. Davis, Gabriel K. Adzika, Alyssa N. Bebenek, Daniel D. Bazianos, Beishan Chen, Elizabeth Mercado-Ayon, Liam P. Flatley, Arjun P. Suryawanshi, Isabelle Ho, Joshua D. Rabinowitz, Suraj D. Serai, David M. Biko, Jaclyn Tamaroff, Anna DeDio, Kristin Wade, Kimberly Y. Lin, David J. Livingston, Shana E. McCormack, David R. Lynch, Joseph A. Baur

**Affiliations:** 1 Department of Physiology, Perelman School of Medicine, University of Pennsylvania, Philadelphia, Pennsylvania, USA.; 2 Division of Neurology, Children’s Hospital of Philadelphia, Philadelphia, Pennsylvania, USA.; 3 Department of Chemistry, Princeton University, Princeton, New Jersey, USA.; 4 Perelman School of Medicine, University of Pennsylvania, Philadelphia, Pennsylvania, USA.; 5 Department of Radiology and; 6 Division of Endocrinology and Diabetes, Children’s Hospital of Philadelphia, Philadelphia, Pennsylvania, USA.; 7 Division of Pediatric Endocrinology, Vanderbilt University Medical Center, Nashville, Tennessee, USA.; 8 Division of Pediatric Cardiology, Children’s Hospital of Philadelphia, Philadelphia, Pennsylvania, USA.; 9 Department of Pediatrics, Perelman School of Medicine, University of Pennsylvania, Philadelphia, Pennsylvania, USA.; 10 Metro International Biotech, Worcester, Massachusetts, USA,; 11 Department of Neurology, Perelman School of Medicine, University of Pennsylvania, Philadelphia, Pennsylvania, USA.

**Keywords:** Cardiology, Metabolism, Mitochondria, Neurological disorders

## Abstract

Friedreich’s ataxia (FRDA) is a progressive disorder caused by insufficient expression of frataxin, which plays a critical role in assembly of iron-sulfur centers in mitochondria. Individuals are cognitively normal but display a loss of motor coordination and cardiac abnormalities. Many ultimately develop heart failure. Administration of nicotinamide adenine dinucleotide–positive (NAD^+^) precursors has shown promise in human mitochondrial myopathy and rodent models of heart failure, including mice lacking frataxin in cardiomyocytes. We studied mice with systemic knockdown of frataxin (shFxn), which display motor deficits and early mortality with cardiac hypertrophy. Hearts in these mice do not “fail” per se but become hyperdynamic with small chamber sizes. Data from an ongoing natural history study indicate that hyperdynamic hearts are observed in young individuals with FRDA, suggesting that the mouse model could reflect early pathology. Administering nicotinamide mononucleotide or riboside to shFxn mice increases survival, modestly improves cardiac hypertrophy, and limits increases in ejection fraction. Mechanistically, most of the transcriptional and metabolic changes induced by frataxin knockdown are insensitive to NAD^+^ precursor administration, but glutathione levels are increased, suggesting improved antioxidant capacity. Overall, our findings indicate that NAD^+^ precursors are modestly cardioprotective in this model of FRDA and warrant further investigation.

## Abstract 2

Nicotinamide riboside or mononucleotide administration improved survival and cardiac phenotypes in a mouse model of Friedreich’s Ataxia and supported antioxidant metabolism in the heart.

## Introduction

Friedreich’s ataxia (FRDA) is the most common genetic disorder directly affecting mitochondria, with an incidence of roughly 1 in 50,000 people in the United States (1). Although there are rare forms driven by point mutations, the most common cause of FRDA is a biallelic GAA trinucleotide repeat expansion in the first intron of the *Fxn* gene, leading to decreased expression of the iron-sulfur cluster assembly protein frataxin (2–4). This disrupts formation of iron-sulfur clusters, which are necessary for the final assembly of enzymes, including NADH dehydrogenases, and elicits toxic deposition of iron in mitochondria. The result is mitochondrial and metabolic dysfunction that is manifested in neurological, endocrine, orthopedic, and cardiac clinical features (5, 6). The typical age of onset is 10–15 years old, with a life expectancy of about 30 years after the initial diagnosis. The most common causes of early mortality in FRDA are cardiac in nature (6–9). There is no cure for heart disease in FRDA, with the exception of heart transplantation; case reports suggest that postheart transplant survival in FRDA is at least as long as the median for other heart transplant recipients, despite neurologic progression of disease (10–13).

Large-scale efforts to understand the early changes in gene expression and cardiac metabolism underlying different forms of heart failure have highlighted the disruption of normal mitochondrial metabolism (14–21). Nicotinamide adenine dinucleotide-positive (NAD^+^) is a crucial cofactor required for cellular and mitochondrial metabolism; it plays multiple roles in metabolic processes, including serving as the carrier for high energy electrons to support ATP generation. Dysfunction of mitochondrial complexes can affect redox homeostasis, which may further contribute to mitochondrial decline (22, 23). NAD^+^ concentration is decreased in failing human hearts (17), and expression of nicotinamide phosphoribosyltransferase (NAMPT), the rate-limiting enzyme for NAD^+^ synthesis, is downregulated during heart failure and in myocardial ischemic injury (24). NAD^+^ precursors such as nicotinamide riboside (NR) and nicotinamide mononucleotide (NMN), which can bypass NAMPT, are protective in preclinical models of heart failure (24–26). NAD^+^ precursors have also been shown to have therapeutic effects in disorders involving mitochondrial dysfunction, including mitochondrial myopathy in humans, as well as mouse models with mitochondrial defects that are primary or secondary to conditions such as Cockayne syndrome (27–31). Together, these observations have generated considerable interest in the potential for NAD^+^ precursors as treatments for heart failure in humans and in whether NAD^+^ deficiency per se is playing a causal role in mitochondrial dysfunction or other aspects of the pathogenesis of heart disease in FRDA.

Most preclinical studies on cardiac phenotypes related to frataxin loss have employed muscle-specific KO of *Fxn* driven by Cre under the control of the muscle creatine kinase (MCK) promoter (Fxn-KO) mice. These mice lack frataxin in both skeletal muscle and cardiomyocytes but progress from hypertrophy to fatal dilated cardiomyopathy at about 10 weeks of age, before any skeletal muscle defect becomes apparent (32). Heart failure in these mice is accompanied by mitochondrial dysfunction, reduced ATP levels, and a skewing of the NAD^+^/NADH redox ratio toward NADH (33–35). In addition, there is strong hyperacetylation of mitochondrial proteins, which has also been observed in other forms of heart failure and may reflect decreased activity of the NAD^+^-dependent mitochondrial deacetylase SIRT3 (17, 35). However, there are likely additional contributing factors since complete KO of SIRT3 causes less severe hyperacetylation than Fxn-KO, and it should be noted that recent work indicates that the hyperacetylation of mitochondrial proteins per se is not causal in heart failure (36). There are conflicting reports indicating a deficit (33, 34) or no change (27) in total tissue NAD^+^ content in Fxn-KO hearts, and whether loss of frataxin decreases NAD^+^ in humans is unknown. Administration of the NAD^+^ precursor NMN improved heart function in Fxn-KO animals, suggesting that NAD^+^ metabolism may influence cardiac pathogenesis in FRDA (27). Furthermore, Fxn-KO hearts had increased expression of *Nmrk2,* the gene encoding NR kinase 2, which provides an alternative route to synthesize NAD^+^ from NR and NMN independently of NAMPT (37). Administration of an alternative NAD^+^ precursor, 6-methoxy-2-salicylaldehyde nicotinoyl hydrazone, decreased iron deposits and modestly increased NAD^+^ in Fxn-KO hearts but did not prevent hypertrophy or fibrosis (33). Overall, there is strong evidence for perturbation of NAD^+^/NADH in Fxn-KO mice and some evidence for benefits of NAD^+^ supplementation, but rescuing phenotypes in these mice is challenging due to the rapid progression and lethality in the complete absence of frataxin.

We set out to explore the potential therapeutic effects of NR and NMN in a progressive mouse model of FRDA with systemic reduction of frataxin and a milder cardiac phenotype with prolonged survival as compared with KO models (38, 39). We found that NAD^+^ levels were lower in male hearts and that NAD^+^ precursor treatment modestly altered the progression of cardiomyopathy and improved overall survival. Although a causal link has not been demonstrated, these observations were associated with an increase in glutathione, which serves as a major defense against iron toxicity and reactive oxygen species (ROS) and may thereby contribute to the overall improvement in the mice.

## Results

To examine the effects of NAD^+^ precursors on the development and progression of FRDA-like pathology in mice, we employed the shFxn transgenic mouse model, a doxycycline-inducible Tet-on system driving an shRNA that silences frataxin mRNA throughout the entire body (38). Adult mice that start doxycycline at 12–16 weeks of age develop multiple pathologies consistent with FRDA, including loss of motor coordination, cerebellar degeneration, reduced muscle mass and strength, and cardiac hypertrophy over the next 12–24 weeks (39, 40). We administered NR or NMN in the drinking water beginning simultaneously with doxycycline treatment.

### Sexually dimorphic survival and cardiac phenotypes in shFxn mice.

**Figure 1:**
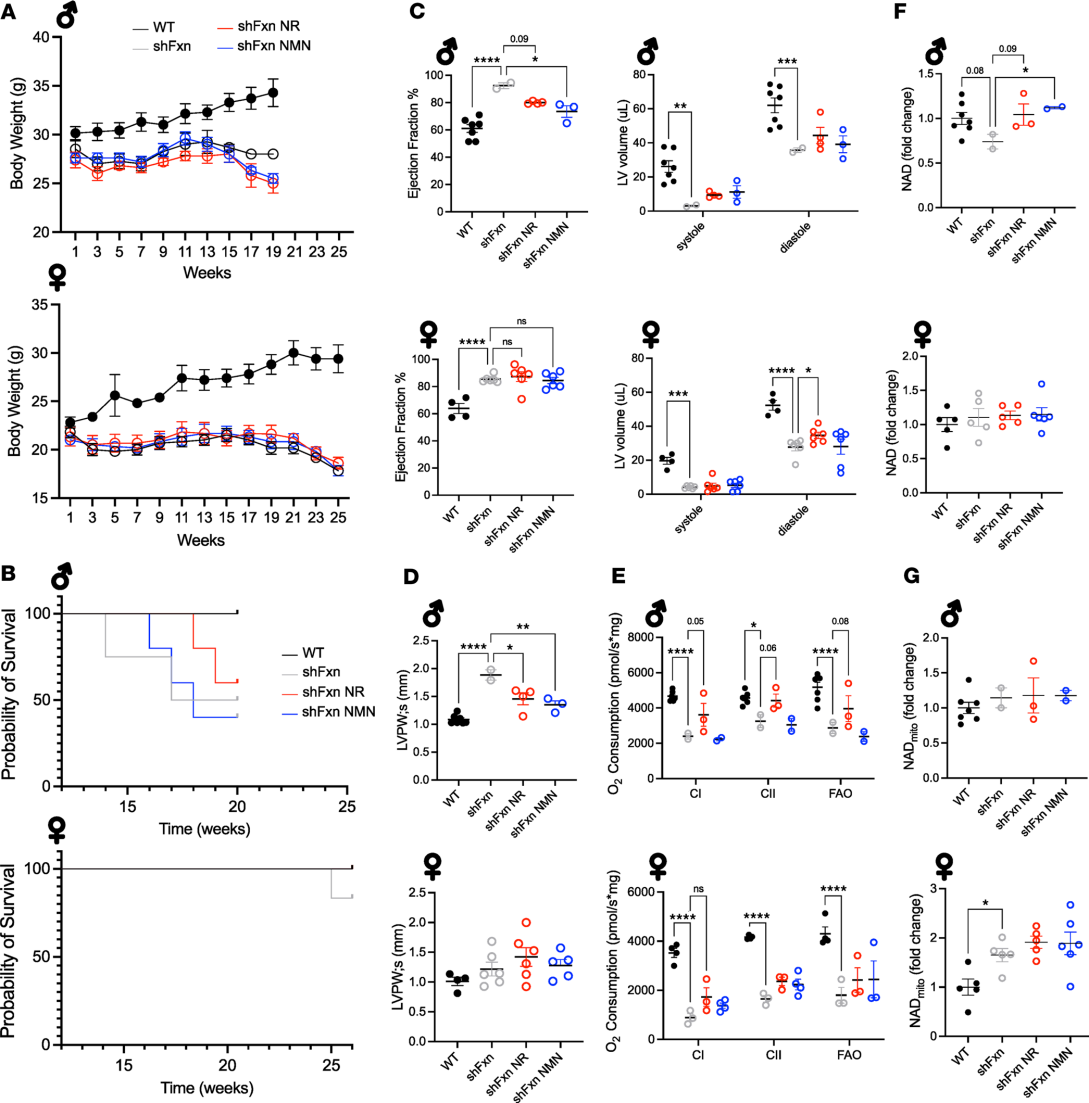
Sexual dimorphism in survival and treatment responses in the shFxn mouse model. (**A**) Body weights of male (top) and female (bottom) shFxn mice following the initiation of doxycycline at 12–16 weeks of age (*n* = 4–7). (**B**) Survival curves for each sex after doxycycline (*n* = 4–7). (**C**) Ejection fraction recorded at 18 weeks after doxycycline and LV volumes at end systole and diastole (*n* = 2–7). (**D**) Systolic LV posterior wall measurements (*n* = 2–7). (**E**) Respirometry in isolated heart mitochondria from males (20 weeks after doxycycline) and females (26 weeks after doxycycline) (*n* = 2–7). (**F**) Heart tissue NAD^+^ (*n* = 2–7). (**G**) Heart mitochondrial NAD^+^ (*n* = 2–7). **A** and **E**, 2-way ANOVA; **B**, Mantel-Cox test for survival; **C**, **D**, **F**, and **G**, 1-way ANOVA (*P ≤ 0.05, **P ≤ 0.01, ***P ≤ 0.001, ****P ≤ 0.0001).

Because no overt phenotypic differences between sexes were previously noted for the shFxn mice, we initially studied mixed cohorts of males and females. Mice were continuously administered chow with doxycycline (200 ppm) and given regular drinking water or drinking water containing NR (3.00 mg/mL) or NMN (3.45 mg/mL, equimolar with NR), doses that we have previously found to be well tolerated and efficacious (41–43). Both male and female shFxn mice failed to gain weight after the initiation of doxycycline and began losing weight by ~12–13 weeks (Figure 1A). By as early as 6 weeks after doxycycline, both sexes had decreased motor coordination, as reflected by reduced latency to fall on the rotarod apparatus (Supplemental Figure 1A; supplemental material available online with this article; https://doi.org/10.1172/jci.insight.177152DS1). Neither weight nor motor coordination was affected by NAD^+^ precursor administration. Because FRDA is associated with an increased risk of diabetes (44), we examined whether shFxn mice exhibited insulin resistance or glucose intolerance. Although glucose was slightly elevated in male shFxn mice after fasting and 4 hours of refeeding, and although this was prevented by NMN, the response to glucose challenge was not significantly affected (Supplemental Figure 1, B and C). Similarly, insulin levels were not significantly changed by frataxin loss or either treatment (Supplemental Figure 1C). Overall, glucose phenotypes were mild with no overt diabetes and were not consistently changed by NR or NMN. Most strikingly, obvious sex differences in overall survival began to emerge by 18 weeks after doxycycline, at which time 50% of untreated male shFxn mice had died, but all female mice survived (Figure 1B). Remaining males were sacrificed at 20 weeks for tissue-level analyses, and the female shFxn mice were maintained until 26 weeks, when the first death occurred. In both sexes of shFxn mice, the LV volume was substantially reduced and the LV was hyperdynamic, with an ejection fraction (EF) over 80% in females and ~90% in males (Figure 1C). Both NR and NMN reduced the elevated EF in males but not in female mice. At 18 weeks after doxycycline, we found sexually dimorphic effects on cardiac hypertrophy. In males, the left ventricular posterior wall (LVPW) was significantly thicker than in WT mice, and this was reduced by both NR and NMN (Figure 1D). However, females exhibited milder and nonsignificant changes in LVPW thickness. At the time of sacrifice, heart mitochondrial respiration was impaired in both sexes (Figure 1E). NR appeared to have a protective effect in males, although the study was underpowered at this point due to the high male mortality. Male shFxn mice trended toward lower NAD^+^ levels in the heart tissue, with NR tending to increase and NMN significantly increasing NAD^+^. At 26 weeks, the female mice did not have decreased NAD^+^ despite the longer period of frataxin deficiency. Mitochondrial NAD^+^ was unchanged in males and higher in shFxn females (Figure 1, F and G). Overall, this pilot study indicated that shFxn mice have sexually dimorphic phenotypes that preclude analyzing males and females together for cardiac parameters or mortality.

### Hyperdynamic hearts in humans with FRDA.

**Figure 2:**
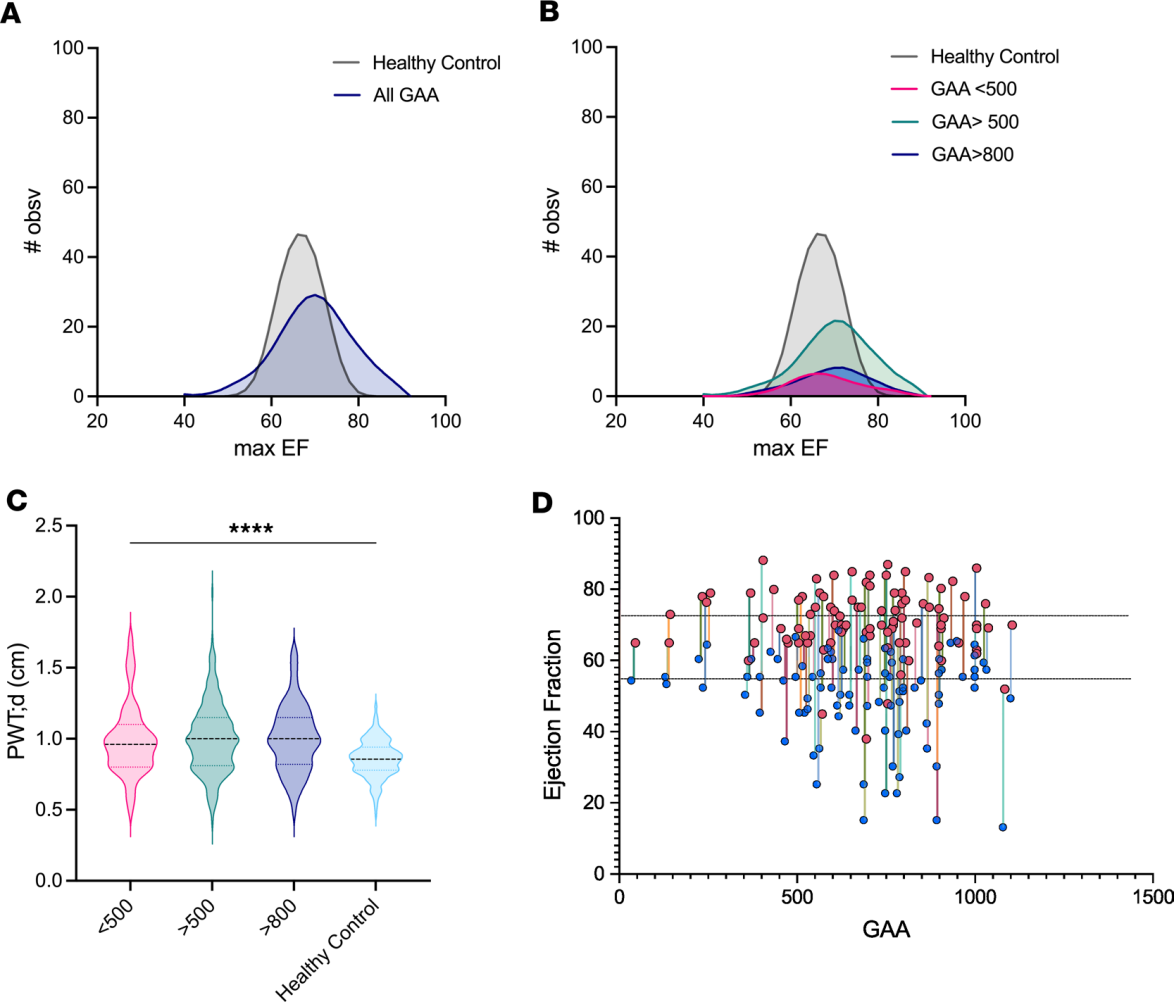
Individuals with higher GAA repeat length are more likely to experience higher maximal EF. (**A** and **B**) Frequency distribution of maximal EFs sorted by GAA (GAA1) repeat length on the shorter allele (*n* = 315). Curves generated by LOWESS regression with coarse smoothing on frequency distribution of maximum EF (bin size = 2). Simulated healthy control data generated by setting mean and SD with gaussian frequency based on ref. 56. (**C**) Posterior wall thickness at diastole at maximal EF (*n* = 315) compared with simulated healthy control data (57). (**D**) Individual patient EF range recorded throughout 6+ years sorted by GAA repeat length (*n* = 106). Peak EF is noted with pink circles, and minimum EF is noted with blue circles. Dotted lines represent minimum and maximum observed EFs in adolescents.

End-stage heart failure in individuals with FRDA is characterized by a gradual loss of EF (1, 6, 8, 9, 45–53). Thus, the observation that shFxn mice have hyperdynamic hearts initially suggested that they might not model cardiac abnormalities in human FRDA, despite their hypertrophic phenotype. However, the natural history of heart failure in FRDA is poorly understood, and we considered the possibility that EF might be elevated earlier in the course of disease. To test this hypothesis, we used data available from the Friedreich’s Ataxia Clinical Outcome Measures Study (FACOMS) and the CHOP Cardiac Records Study (54, 55), which includes medical records for 406 participants and over 2,500 echocardiograms. Ninety-one individuals did not have EF recordings or had initial recordings that were already in later-stage cardiomyopathy with loss of systolic function (EF < 50%). From the remaining 315 participant records, we found that EFs frequently exceeded the range of normal values for healthy controls at the same age (primarily adolescents) (56). Moreover, measurements from individuals with higher GAA repeat length on the shorter allele (GAA1) (>500, indicative of more severe disease) had a frequency distribution favoring higher EFs compared with those from individuals with lower GAA1 repeat count (<500) (Figure 2, A and B). Further restricting the analysis to GAA1 > 800 did not meaningfully change the EF distribution. Similarly, diastolicposterior wall thickness (PWT) recorded at the peak EF visit in individuals with FRDA was increased compared with the range reported for healthy controls at 24 years old (57) (Figure 2C). We then examined the range of EF values in a subset of the 406 individuals with FRDA that had multiple longitudinal echocardiographs over at least 6 years (*n* = 106), finding that a wide range of values were typical within a given individual (Figure 2D). Few data were available for males over the age of 40, and no significant differences in EF were observed between sexes under the age of 40. We also found that peak EFs over 72% (the approximate limit for normal controls) were recorded at younger ages, whereas peak EFs within the normal range were more widely distributed (Supplemental Figure 2A). While it is clear that EF decreases over time and can drop precipitously in failing hearts, these data demonstrate that many individuals with FRDA experience an elevated EF early in the course of disease (Supplemental Figure 2, B and C). The increased EF in shFxn mice may be reflective of similar changes and, thus, provide important clues as to the origins of the eventual heart failure in humans with FRDA.

### NAD^+^ administration improves survival of male shFxn mice.

**Figure 3:**
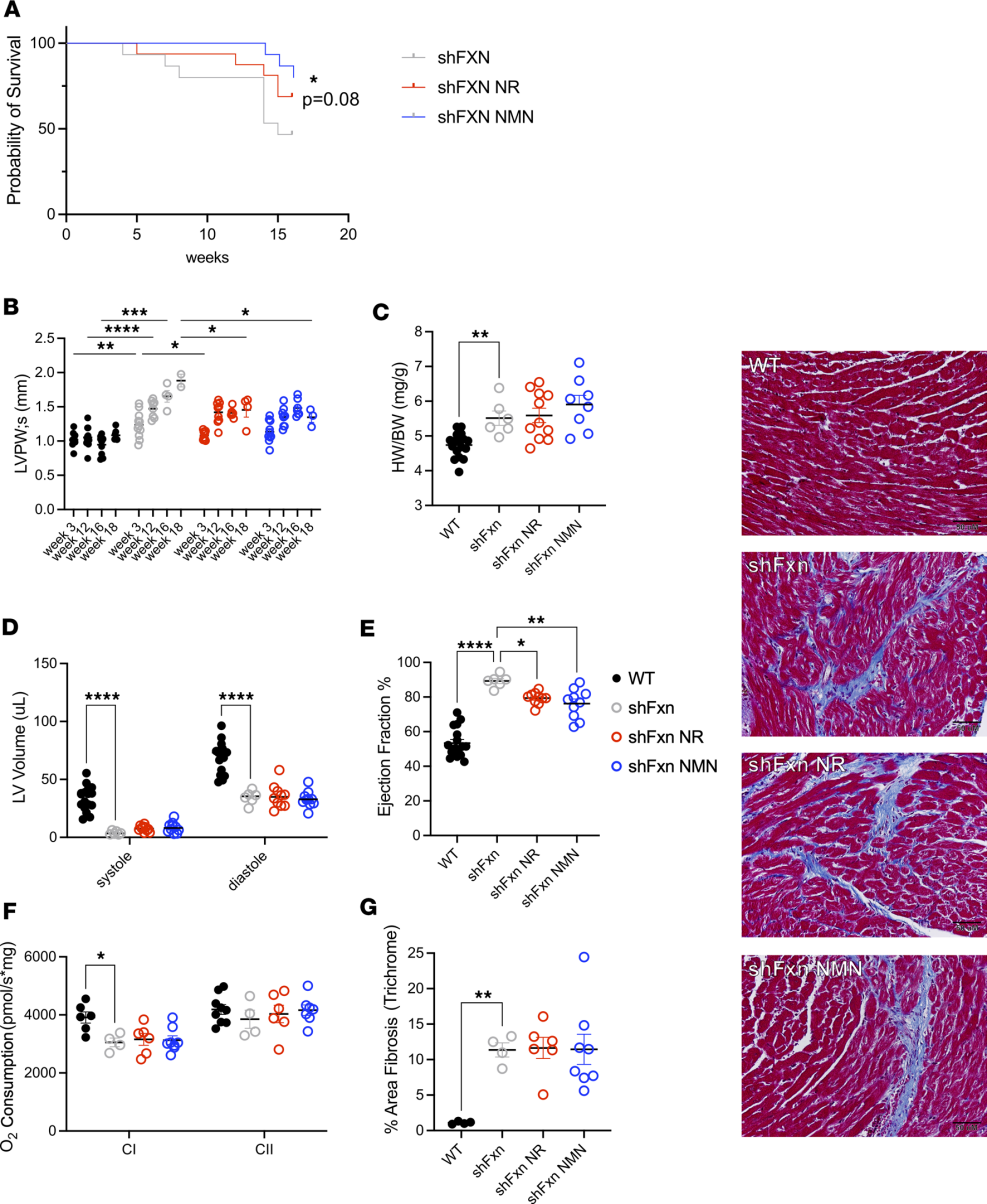
NR and NMN improve survival and heart function in male shFxn mice. (**A**) Male shFxn mice treated with NAD^+^ precursors have significantly longer survival compared with nontreated mice. (**B**) NAD^+^ precursors significantly reduce LV posterior wall thickness at 16–18 weeks after doxycycline. (**C**) shFxn mice have increased heart weight relative to body weight at 16–18 weeks. (**D** and **E**) LV systolic and diastolic volumes and EF after 16–18 weeks measured by echocardiography. (**F**) Mitochondrial complex I respiration is modestly decreased in 16-week shFxn hearts. (**G**) Masson’s trichrome blue staining of LV section. Staining and quantification shows shFxn mice have fibrosis in hearts that is not reduced by treatment. Scale bar: 50 μM. **A**, pairwise Mantel-Cox test for survival vs. untreated, *n* = 10–11; **B**, **D**, and **F**, *n* = 4–12, 2-way ANOVA; **C**, **E**, and **G**, *n* = 4–12, 1-way ANOVA (*P ≤ 0.05, **P ≤ 0.01, ***P ≤ 0.001, ****P ≤ 0.0001).

Due to the stronger phenotypes and responses to treatment, but low numbers in the male groups of shFxn mice, we performed a follow-up study using all-male cohorts. As in the previous study, untreated male shFxn mice exhibited early mortality, with just under half of the mice surviving to 16 weeks after frataxin knockdown. Encouragingly, administration of NAD^+^ precursors prolonged survival in shFxn mice (Figure 3A and Supplemental Figure 3A), with NMN alone or pooled data reaching statistical significance.

### Administration of NAD^+^ precursors modestly improves cardiac phenotypes in male shFxn mice.

We evaluated heart function by echocardiography beginning at 3 weeks after doxycycline. By this time, the EF in male shFxn mice had already increased from ~50% to ~65%, and it continued to increase throughout the course of the experiment (Supplemental Figure 3B). When evaluated at 16–20 weeks, male shFxn mice had hyperdynamic LV function, with an average EF of almost 90%, and LVPW thickness had nearly doubled (Figure 3, B and E, and Supplemental Figure 3B). Mice receiving NR or NMN exhibited a significantly smaller increase in LVPW thickness, in agreement with our results from the first cohort (~1.4 mm, while some nontreated animals reached nearly 2 mm, *P*_NR_ < 0.0002, *P*_NMN_ = 0.0003) (Figure 1D and Figure 3B). Despite the decreased wall thickness, heart weight/body weight ratio was not decreased by NR and NMN (Figure 3C). The LV volume was decreased in all shFxn mice and not significantly changed by NR or NMN treatment (Figure 3D); however, NAD^+^ precursors limited the increase in EF to under 80% (vs. > 90% in nontreated mice, *P*_NR_ = 0.01, *P*_NMN_ = 0.001) (Figure 3E). Brain natriuretic peptide (BNP) levels, which can indicate heart failure (58), were unchanged by frataxin depletion or NAD^+^ precursors (Supplemental Figure 3C). Mitochondrial respiration was diminished by about 25% in shFxn mice at 16 weeks after doxycycline in the heart (Figure 3F) but not in the liver (Supplemental Figure 3D). Mitochondrial dysfunction was milder than in the previous experiment in which mice had been sacrificed at 20 weeks after doxycycline (Figure 1E), suggesting that this phenotype may have a late onset relative to other cardiac changes. There was no appreciable improvement in mitochondrial respiration in the NR- or NMN-treated groups. Staining with Masson’s trichrome blue revealed markedly increased fibrosis in the shFxn mice, which was not affected by NR or NMN (Figure 3G). Overall, male shFxn mice exhibited consistent thickening of the LVPW, a drop in LV chamber size, and an increase in EF that is likely compensatory. Treatment with NR or NMN had only modest protective effects but seemed to limit the peak values for both the LVPW thickening and EF increases.

### Transcriptional changes in hearts of shFxn mice.

**Figure 4:**
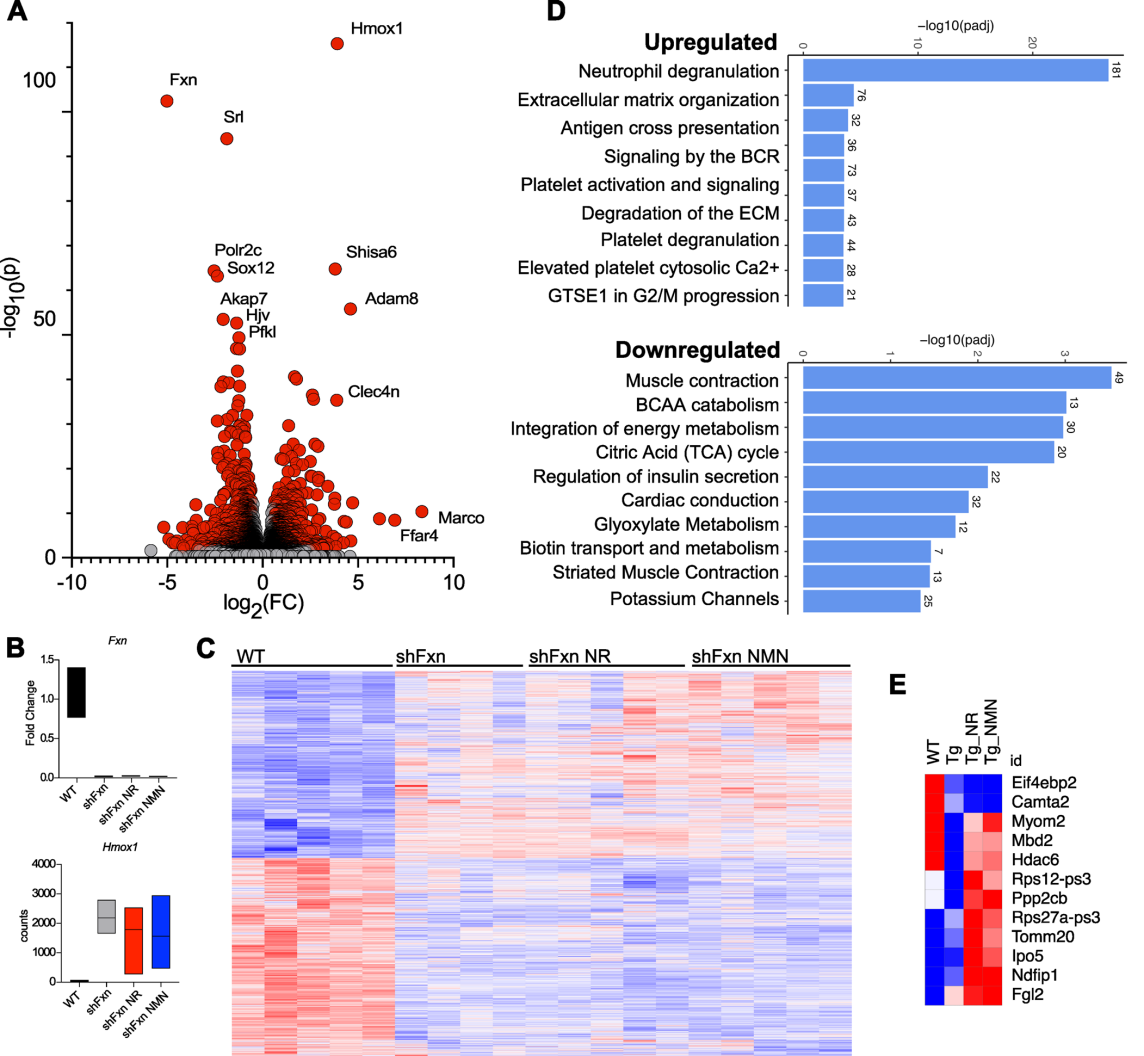
shFxn hearts exhibit an mRNA signature of inflammation that is not affected by NAD^+^ precursor treatment. (**A**) Volcano plot indicating upregulated and significantly downregulated genes in heart tissue of shFxn male mice at 16 weeks after doxycycline. Red are significantly upregulated or downregulated (*P*_adj_ ≤ 0.05, FDR-adjusted Wald test). (**B**) *Fxn* is not recovered by precursor treatment (mean, lower, upper bound; WT: 1.085, 0.763, 1.402; shFxn: 0.019, 0.006, 0.025; NR: 0.021, 0.016, 0.030; NMN: 0.017, 0.012, 0.020), *Hmox1* is highly induced in shFxn mice (WT: 151.6, 115, 196; shFxn: 2,303, 1,773, 2,909; NR: 1,906, 387, 2,649; NMN: 1,677, 581, 3,063). (**C**) Heatmap of all transcripts demonstrating minor transcriptional changes with treatment. (**D**) Upregulated and downregulated reactome terms in shFxn mice. (**E**) Subset of genes that are modified by NR and NMN.

To gain insight into the mechanisms mediating cardiac phenotypes in the shFxn mice, we performed RNA-Seq on hearts (GEO accession no. GSE271798). Frataxin loss led to dramatic transcriptional changes (Figure 4A; *P*_adj_ ≤ 0.05). Heme oxygenase 1 (*Hmox1*), a target of NRF2, was the most highly significantly upregulated gene, whereas the macrophage-associated genes *Marco* and the free fatty acid receptor *Ffar4* exhibited higher fold-changes but more variability (Figure 4, A and B). Neither NR nor NMN treatment affected *Fxn* mRNA level in the heart (Figure 4B) or had a strong influence on transcriptional profile overall (Figure 4C and Supplemental Figure 4A). Immune upregulation was a prominent feature of shFxn hearts but was generally unaffected by NR or NMN (Figure 4D and Supplemental Figure 4A). Notably, it is unclear in some cases whether transcripts reflect infiltrating immune cells or are expressed by cardiomyocytes. Enrichment analysis identified neutrophil degranulation as the most highly upregulated pathway, whereas terms related to muscle contraction and energy metabolism were downregulated in shFxn mice (Figure 4D). Genes associated with cardiomyocyte fibrosis were also highly enriched in shFxn mice and were unchanged with NR or NMN treatment (Supplemental Table 1), consistent with our trichrome staining. Analysis of male and female hearts saved from our pilot experiment revealed largely consistent changes in females, with the exception of a notable upregulation of genes related to metabolism of RNA and RNA processing (Supplemental Figure 4, B and D).

Given the limited influence of NR and NMN on transcriptional profiles, we next performed a more focused search for consistent transcriptional changes between the 2 treatments (Figure 4E). Using combined information from hearts of NR- and NMN-treated mice, we found a small list of genes that were recovered with treatment, including *Myom2* (M-protein) and *Hdac6*. M-protein is part of the 3D stabilization of the M-band in the sarcomere and binds to titin. *Hdac6* has previously been reported to influence cardiac contractility, potentially by modifying the elasticity of tubulin through deacetylation (59). Otherwise, there were few transcriptional changes in NR- and NMN-treated mice that could provide a straightforward explanation for their protective effects on the heart or overall survival.

### Loss of frataxin induces major changes to the metabolome.

**Figure 5:**
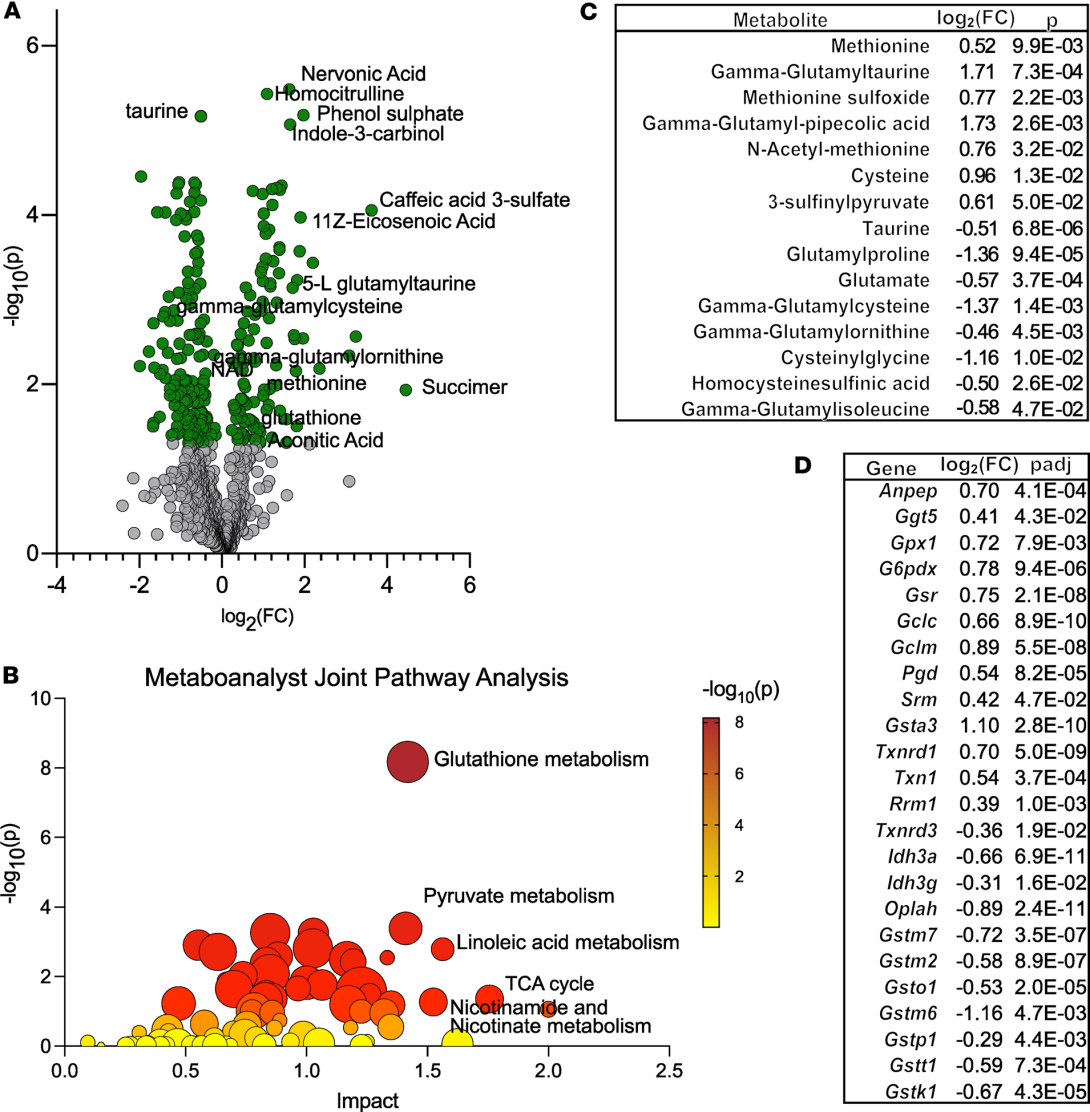
Joint pathway analysis identifies glutathione metabolism as highly affected in shFxn hearts. (**A**) Volcano plot indicating significantly depleted and enriched metabolites in shFxn heart tissue (*P* ≤ 0.05, 2-tailed *t* test). (**B**) Metaboanalyst joint pathway analysis using combined transcript data (*P*_adj_ ≤ 0.05, Wald test, FDR-adjusted) and metabolite data (*P* ≤ 0.05, 2-tailed *t* test). (**C**) Glutathione synthesis–related metabolites modified in shFxn hearts. (**D**) Glutathione synthesis and function-related genes modified in shFxn hearts.

To better understand the molecular changes induced by frataxin loss and NAD^+^ precursor administration, we next performed untargeted metabolomics on heart tissue, plasma, heart mitochondria, and cerebellum (Figure 5A, Supplemental Figure 5A, and Supplemental Table 2A) (Metabolights, accession no. MTBLS10625). Loss of frataxin dramatically altered the metabolomes, with changes skewed toward increasing metabolites in the plasma, cerebellum, and heart mitochondria and more balanced increases and decreases in heart tissue (nominal *P* ≤ 0.05). Pathway analysis performed in Metaboanalyst using all nominally significant metabolite changes (*P* ≤ 0.05, FDR < 0.2) after loss of frataxin revealed effects on arginine biosynthesis, alanine metabolism, TCA cycle, taurine metabolism, and glutathione metabolism with many of the changes common among the heart, cerebellum, and mitochondria (Supplemental Table 2A). The most extensive changes occurred in the heart tissue, with further effects on tryptophan metabolism and tRNA biosynthesis. Although arginine and alanine-related metabolites were significantly increased in the heart tissue, heart mitochondria, and cerebellum (Supplemental Table 2B), these may reflect systemic effects since many of the same metabolites were also changed in the plasma. A subset of TCA metabolites were significantly changed in heart tissue, and branched chain amino acids (BCAAs) were depleted, consistent with enrichment for BCAA metabolism in transcriptional analyses (Supplemental Figure 5B).

### Glutathione synthesis is highly upregulated in hearts of shFxn mice.

To take advantage of our combined data sets, we performed joint pathway analysis on metabolomic and transcriptomic changes using Metaboanalyst (Figure 5B). The combined data sets strongly implicated changes in glutathione metabolism, in addition to smaller signals for pyruvate metabolism, TCA cycle, and nicotinamide metabolism in the hearts of shFxn mice (Supplemental Table 2C). The transsulfuration pathways and the γ-glutamyl transferase pathways are related to glutathione synthesis, and several metabolites and genes in these pathways were also modified by frataxin loss in the heart tissue (Figure 5, C and D).

### NAD^+^ levels are reduced in male shFxn mice.

**Figure 6:**
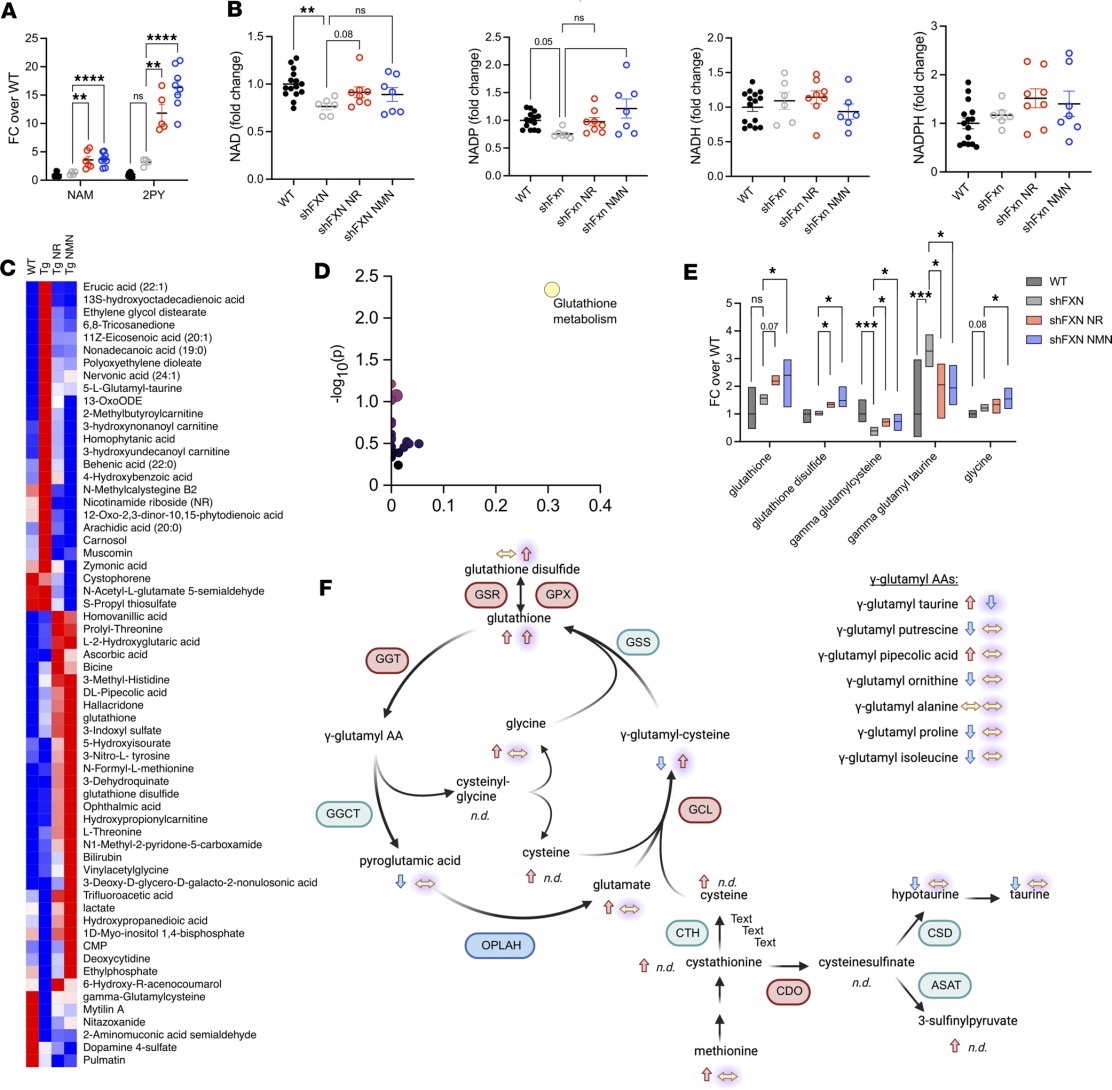
NAD^+^ precursor treatment amplifies glutathione synthesis in shFxn hearts. (**A**) Plasma nicotinamide and 2-PY levels are increased in treated mice. (**B**) NAD^+^ and NADP^+^ are significantly depleted in shFxn hearts, with recovery of NADP with NMN treatment. NADH and NADPH levels are unchanged with loss of frataxin and with precursor treatment. (**C**) Heatmap of metabolites significantly changed with NR and NMN treatment (pooled analysis). Bolded metabolites were significantly modified by depletion of frataxin. (**D**) Using list in **C**, Metaboanalyst identified glutathione metabolism as significantly changed by treatment in shFxn hearts. (**E**) Glutathione related metabolites are altered in shFxn mice and treated mice. [Box Plots: Metabolite-[group:mean, lower, upper]] [Glutathione-[WT:1, 0.46, 1.97; shFxn:1.57, 1.33, 1.71; NR:2.189, 2.04, 2.39; NMN:2.40, 1.24, 2.97] Glutathione Disulfide-[WT:1, 0.68, 1.16; shFxn:1.02, 0.95, 1.11; NR:1.35, 1.23, 1.43; NMN:1.49, 1.25, 2.00] γ-glutamylcysteine-[WT:1, 0.71, 1.52; shFxn:0.39, 0.22, 0.53; NR:0.72, 0.54, 0.83; NMN:0.72, 0.39, 1.00] γ-glutamyltaurine-[WT:1, 0.17, 2.97; shFxn:3.27, 2.70, 3.87; NR:2.05, 0.84, 2.81; NMN:1.95, 1.32, 2.77] Glycine-[WT:1, 0.86, 1.14; shFxn:1.21, 1.09, 1.35; NR:1.34, 1.02, 1.55; NMN:1.54, 1.185, 1.94]. Brackets indicate pairwise comparisons with *P* values indicated for several that did not reach significance. (**F**) Model representing metabolite changes in glutathione synthesis pathways. Left arrow is direction in shFxn vs. WT mice, right arrow with glow is direction in shFxn vs. treated mice, red symbols are upregulated in shFxn mice, blue symbols are downregulated, and green are unchanged. **A**, **B**, and **E**, *n* = 6–17, 1-way ANOVA, Fisher’s LSD test); **C**, *n* = 6–17, normalized ion value, 2-tailed *t* test (*P ≤ 0.05, **P ≤ 0.01, ***P ≤ 0.001, ****P ≤ 0.0001).

As expected, the plasma levels of nicotinamide and its methylpyridone metabolites were dramatically increased after administration of NR and NMN (Figure 6A). There are conflicting reports on whether complete loss of frataxin depletes NAD^+^ in hearts, and there are no prior reports of this phenomenon in the shFxn mouse model. In the combined set of male shFxn mice (16–20 weeks after doxycycline), both NAD^+^ and NADP^+^ were decreased significantly (Figure 6B). Interestingly, no such decline was noted in female hearts, despite an extra 6 weeks of treatment (Figure 1). We also measured NAD^+^ in isolated cardiac mitochondria and found no change, even in animals with lower tissue NAD^+^ levels (Supplemental Figure 6A). We also did not observe a difference in cerebellar tissue NAD^+^ levels (Supplemental Figure 6B). NR did not completely restore NAD^+^ or NADP^+^ in the heart but trended toward increasing both (*P* = 0.08, *P* = 0.09, respectively), and NMN significantly restored NADP^+^ (*P* < 0.01) (Figure 6B). The NAD^+^/NADH ratio was also decreased in shFxn mice (Supplemental Figure 7A), but not significantly modified by NAD^+^ precursors. Overall, these findings suggest that a decrease in NAD^+^ concentration correlates with the worse phenotype and clearer treatment responses in male as compared with female shFxn mice. NR and NMN had only minor effects on metabolites related to de novo nucleotide synthesis (Supplemental Figure 7B).

### NAD^+^ precursors increase glutathione synthesis.

NR or NMN treatment affected a large number of metabolites but did not consistently reverse the effects of frataxin loss in the heart or other metabolomes. Heart tissue was the most significantly altered by treatment (Figure 6C and Supplemental Figure 6, A–C), whereas in cerebellum, mitochondria, and plasma, there were comparatively few changes with NR and NMN (Supplemental Figure 6, A–C). We compared nominally significant changes with NR or NMN treatment in the heart (Supplemental Table 3), as well as changes with both treatments considered as a combined group (Figure 6C). Of the latter, about one-third reflected restoration of metabolite levels that had been altered in shFxn compared with WT. Several of these were free fatty acids and related molecules. Lactate was depleted in shFxn mice and increased above WT levels with NR and NMN. γ-Glutamylcysteine was also depleted in shFxn mice and partially restored by NAD^+^ precursor treatment. Pathway analysis using metabolites altered by NAD^+^ precursor treatment revealed a strong connection to glutathione metabolism, driven primarily by the increases in glutathione, glutathione disulfide, and γ-glutamylcysteine (Figure 6, D–F). Overall, these trends were suggestive of treatment-driven increases in glycolysis and glutathione metabolism.

NRF2 is a major stress-responsive transcription factor that upregulates the synthesis of multiple antioxidant pathways, including glutathione synthesis, and is the target for the only approved drug to treat FRDA, omaveloxolone (51, 52). Activation of NRF2 in the shFxn hearts was also suggested by upregulation of *Hmox1*. We reexamined our RNA-Seq data for NRF2 targets and found that a number were increased in the untreated shFxn mice (Supplemental Figure 7C), including genes necessary for glutathione synthesis. Expression of the gene encoding the cystine transporter *Slc7a11*, an important mechanism for intracellular cysteine maintenance, was increased more than 10-fold. mRNA expression of a subset of NRF2 targets involved in glycolysis was reduced (Supplemental Figure 7C), consistent with the observation of low lactate levels in untreated shFxn mice. In contrast to the more widespread effects of frataxin loss on NRF2 targets, the effects of NR and NMN treatment were mainly restricted to the glutathione synthesis pathway, suggesting that they might enhance an adaptive response to increase this antioxidant, which could lessen oxidative damage that occurs in FRDA (60, 61).

## Discussion

We sought to address the potential benefit of NAD^+^ supplementation on cardiac phenotypes related to FRDA using the knockdown model of Chandran et al., in which doxycycline-inducible expression of a short hairpin RNA suppresses frataxin throughout the body (shFxn) (38). It was previously shown that shFxn mice develop progressive cardiac hypertrophy of the left ventricular wall, which is reminiscent of the early cardiomyopathy that develops in humans with FRDA (38, 62). This model is advantageous in that it lowers frataxin throughout the body, which mimics the clinical condition better than a complete KO, and it develops pathology more quickly than models that retain more frataxin, such as knockin/KO (KIKO) mice developed by Miranda et al. in 2002 (63). At the same time, shFxn mice bypass any developmental issues that might be experienced in humans and have lower frataxin expression than individuals with FRDA, contributing to faster and potentially altered disease progression.

Our data suggest a benefit for NAD^+^-based therapeutics in the shFxn model, including an overall increase in survival among males. Although we cannot definitively assign a cause of death in these mice, the improvements in survival were associated with modest improvements in cardiac phenotypes. NR or NMN administration had little effect on early cardiac abnormalities but limited the most extreme increases in left ventricular wall thickness and EF. Unlike humans with FRDA or Fxn-KO mice, male shFxn mice maintain increased LV wall thickness and high EFs that reach over 90% until death (Figure 1C and Figure 3E). Our results are directionally similar, but more severe in terms of both cardiac phenotypes and mortality than those of Vásquez-Trincado, despite similar doxycycline dosing regimens (39). We considered the possibility that early hypercontractility preceded decompensation. However, we never observed declining EFs, even when recording the day before spontaneous death. We were also unable to demonstrate an association between the longitudinal EF measurements and mortality, but we note that our time resolution and number of animals limited statistical power for this test. We suspect that the dramatically reduced diastolic LV volume likely drives the EF very high to maintain cardiac output. Increased heart rate could also contribute to the maintenance of cardiac output; however, we were not able to fully assess this because the presence of anesthesia artificially lowers heart rate measures during echocardiography. The lack of classic signs of heart failure at death suggests that the increased wall thickness combined with extremely high EFs might increase the likelihood of a lethal cardiac event, such as an arrhythmia, or that the deaths are noncardiac. In support of the former possibility, female mice have less severe cardiac pathology and survive considerably longer. Moreover, preventing the most severe cardiac phenotypes in males with NAD^+^ precursors correlates with improved survival but has no effect on motor coordination.

Interestingly, the more severe cardiac phenotypes in male mice are associated with a decrease in heart NAD^+^ concentration that is not seen in females, and only males showed a measurable improvement with NAD^+^ precursor administration. The gene that encodes NRK2, a kinase that supports NAD^+^ synthesis from NR, is upregulated in hearts from both sexes of shFxn mice but to a greater degree in females at the time points observed. This might contribute to their more successful NAD^+^ maintenance (Supplemental Figure 4C) and perhaps survival, although it remains unclear whether NR is available to cardiomyocytes in the absence of exogenous administration. Additional functions for NRK2 have been considered based on its integrin-like structure, which might include modification of laminin deposition in the basal lamina of cardiomyocytes (64).

Because shFxn mice experienced increased EF rather than the reduced EF associated with end-stage heart failure, we turned to a natural history study of humans with FRDA to ask whether this might be relevant to earlier stages of the disease. Data from FACOMS show that the distribution of peak EFs in all individuals or the subset with GAA1 repeat > 500 is substantially higher than that for healthy controls. Furthermore, the highest EFs occurred at younger ages, when those with more severe disease are diagnosed. We also confirmed that individuals with FRDA exhibit thickening of the posterior left ventricular wall, a phenotype that is present in the shFxn mice. These findings suggest that a hyperdynamic LV is a common observation among individuals with FRDA. Our findings are consistent with the EFs recently reported by Pousset et al. (53), who also showed a sex difference in the progression of heart failure in individuals with FRDA. While the heart can become hyperdynamic prior to the loss of EF in other forms of hypertrophic cardiomyopathy, this phenomenon has not been well studied or accepted in FRDA. We suggest that this biphasic change in EF is a feature of FRDA as well and that shFxn mice may serve to model the early cardiac events in severe FRDA.

Mitochondrial dysfunction is a hallmark of FRDA, likely due in part to the primary role of frataxin in the assembly of iron-sulfur clusters needed for the electron transport chain (2, 4, 65, 66). We initially hypothesized that frataxin depletion and the subsequent deleterious effects on mitochondria might lower the mitochondrial NAD^+^ level and that impaired mitochondrial function would be a key driver of heart failure. At 20 weeks in males and 26 weeks in females, mitochondrial respiratory capacity had declined over 50% in complex I and II, but mitochondrial NAD^+^ was maintained in shFxn hearts from both sexes. At 16 weeks, male shFxn mice had only lost about 25% of their capacity for complex I–dependent respiration, suggesting that heart mitochondria decline progressively with a relatively late onset and do not exhibit depletion of NAD^+^. These observations are consistent with a previous report describing mitochondrial deficits at 18 weeks but only minor effects at 10 weeks in cardiac mitochondria from these mice (40). Moreover, mice with deletion of *Fxn* also exhibit a period of normal mitochondrial function in the absence of frataxin (32) and maintain mitochondrial NAD^+^ levels despite mitochondrial dysfunction (35). In contrast to the late-developing mitochondrial defects, cardiac phenotypes clearly emerge within 3 weeks of doxycycline treatment, suggesting that they are likely independent of mitochondrial respiratory capacity. Finally, female shFxn mice at 26 weeks had a greater reduction in mitochondrial function than males at 20 weeks, despite milder cardiac phenotypes and almost no mortality. Thus, mitochondrial dysfunction alone appears unlikely to be the root cause of cardiac phenotypes in shFxn mice, although it is present and may well contribute to the eventual heart failure with reduced EF in patients (14, 16, 67).

We performed RNA-Seq to explore the transcriptional changes occurring in the hearts in the shFxn mice with or without NR and NMN. Both treated and untreated hearts displayed a dramatic inflammatory phenotype with a strong signature of neutrophil degranulation, increased mRNA expression of *Hmox1,* and even stronger but more variable expression of macrophage genes such as *Marco*. This is consistent with observations of inflammation in the hearts of humans with FRDA and a recent study that listed neutrophil degranulation as a top enriched process identified from peripheral blood in humans (68). Downregulated processes included muscle contraction, BCAA catabolism, TCA cycle, cardiac conduction, and potassium channels. A recent paper describing proteomic changes in shFxn mice revealed some consistently modified targets when compared with our transcriptional data, including products of *Ftl1*, *Fth1*, *Srl*, and *Hmox1* (39). Exploring these changes may be useful for identifying biomarkers or new therapeutic targets, but the majority were not meaningfully influenced by NR or NMN treatment. Thus, the influence of NAD^+^ precursor treatments within the heart appears to more likely reflect direct metabolic effects, rather than changes in transcriptional programs.

Metabolomic profiling of hearts from shFxn mice initially pointed to changes in arginine biosynthesis and metabolism of several other amino acids. However, many of these changes were also present in the plasma, suggesting that their origins might have been in other tissues. To gain more precise insight into heart-specific changes, we performed a joint pathway analysis using Metaboanalyst to combine information from our transcriptomic and metabolomic data sets. This analysis identified glutathione metabolism as the most highly altered pathway in shFxn hearts compared with those of WT littermates. Glutathione is a major antioxidant for the cell and is critical to resisting iron-induced damage that is thought to occur as a result of failing to maintain adequate synthesis of iron-sulfur centers. While our standard extraction method did not allow for recovery of cysteine, dedicated analysis of a separate set of shFxn heart tissues revealed a doubling of the abundance of this amino acid, which is a precursor to glutathione and other antioxidants, consistent with our observation that the cystine transporter *Slc7a11* is highly induced in shFxn mice.

NR and NMN had numerous effects on metabolic pathways in shFxn mice but did not consistently oppose the effects of frataxin loss. When all statistically significant metabolites changes in the treated groups compared with the untreated shFxn group were compiled and analyzed, glutathione metabolism was the pathway most affected by NAD^+^ precursor administration. Both glutathione and glutathione disulfide levels were increased by NR or NMN treatment. In addition, γ-glutamylcysteine, the direct precursor to glutathione, was depleted in shFxn mice and partially recovered by NR and NMN. Thus, our data suggest an upregulation of glutathione synthesis in heart tissue that may help control oxidative stress brought on by frataxin loss (69) and that increasing NAD^+^ availability supports increased glutathione synthesis. Notably, glutathione synthesis is an expected result of activation of NRF2, the target of omaveloxolone, which is the only approved therapy for FRDA. Thus, it is possible that NAD^+^ precursors could have an additive or synergistic effect on any glutathione-dependent benefits of this treatment. Encouragingly, NMN was well tolerated by patients with FRDA at 1,000 mg/day and significantly increased NAD^+^ levels in the blood (clinicaltrials.gov, NCT04817111). These results suggest that individuals with FRDA are likely to tolerate NAD^+^ precursors similarly to healthy individuals, and they support more definitive investigations of potential benefits.

### Limitations of the study.

Our study has several limitations. First, because we performed transcriptomic and metabolomic profiling after disease had progressed to ~50% survival in nontreated animals, there is a survival bias in the data, which we would expect to cause underestimation of differences. We also note that several types of controls were possible; we elected to use WT mice treated with doxycycline in order to control for any direct effects of the drug on mitochondria. We did not include uninduced mice carrying the shFxn transgene, which might influence transcription of neighboring genes or cause other unanticipated differences. Encouragingly, both types of controls behaved similarly in the original studies describing this strain (38) and the inflammatory transcriptional signature that we uncovered is similar to that reported in humans with FRDA (68). We were surprised by the difference in disease progression and survival between male and female mice, and we cannot ascertain that the deaths are cardiovascular in nature. No overt sexual dimorphism is observed in end-stage heart failure in patients with FRDA (70). However, the mortality in shFxn mice occurs while their cardiac phenotypes resemble those of patients in the early stages of hypertrophy, prior to decompensated heart failure. Thus, we propose that the deaths most likely reflect arrhythmias as a result of rapid cardiac remodeling and that males are more susceptible to this process. Sexual dimorphism in survival is common in rodent models of heart failure and cardiovascular disease (71–73). For example, transgenic models expressing mutant troponin exhibit sudden cardiac death in males, but not females, reminiscent of our survival data (74). Finally, we note that treatment with NR and NMN was initiated simultaneously with doxycycline, meaning that any benefits that we see should be considered prevention, rather than evidence that established disease can be treated.

### Conclusion.

The consistency between the left ventricular wall hypertrophy and high EF in this mouse model and the cardiomyopathy in the most severely affected patients with FRDA supports the use of this model to explore potential therapies. Overall, our data demonstrate a benefit from of NAD^+^ precursors in mitigating the most severe cardiac phenotypes and early mortality in shFxn mice. We conclude that further exploration of the potential benefits of administering NAD^+^ precursors in individuals with FRDA is warranted.

## Methods

### Sex as a biological variable.

Both male and female mice were examined in this study, and differences in phenotypes are detailed in Figure 1. The sex is indicated accordingly on each figure. After learning of differences in the relevant phenotypes of male and female mice in the first cohort, only male mice were examined.

### Animals.

Inducible Transgenic shFxn mice were originally generated at UCLA (38) and housed at Children’s Hospital of Philadelphia. Mice were bred by crossing C57BL/6J WT mice with transgenic shFxn mice. Loss of *Fxn* mRNA was induced by administering doxycycline chow (5015 base diet plus 200 ppm doxycycline) when the mice were 12–16 weeks of age. NR chloride (3.0 mg/mL) or NMN (3.5 mg/mL) was introduced in the drinking water simultaneously with the start of doxycycline diet. In the first cohort, male and female mice were studied for 20–26 weeks after the initiation of doxycycline. In the second cohort, male mice were studied for 16 weeks after the initiation of doxycycline. Disease progression was monitored by echocardiography as well as assessments of body weight, ataxia, and glucose tolerance as described below. Mice were euthanized by cervical dislocation.

### Echocardiography.

To prepare for echocardiography, mice were anesthetized with 2% isofluorane for 3 minutes, and hair was removed from the ventral thoracic region to expose skin. After 1–2 hours or the following day, echocardiographic scans were performed with mice anesthetized using 2% isofluorane on a heated platform for the duration of the scan. Warmed ultrasound gel was applied to the ventral region, and mice were scanned using an MX500D transducer on a VEVO3100 system (VisualSonics). Images for short axis (SAX) and parasternal long axis (PSLAX) were collected in B-mode and M-mode and analyzed using VevoLab 2.1 and VevoStrain (Visualsonics). In Figure 3E, SAX and PSLAX were averaged to allow the inclusion of animals for which only 1 type of image was of sufficient quality. The corresponding analysis using SAX only to reach a similar conclusion is provided in Supplemental Figure 3, E and F. In the first cohort, male mice were evaluated 1 time at 18 weeks after doxycycline, and female mice were evaluated at 18, 22, and 26 weeks. In the second cohort, male mice were evaluated at 3, 12, and 16 weeks.

### Rotarod evaluation.

Mice were evaluated for development of ataxia by monitoring the latency to fall using a Rotarod Apparatus (Ugo Basile) at 6, 10, 14, and 18 weeks after doxycycline. Mice were initially trained at 3 weeks. They were placed on the rotarod and allowed to sit for 1–2 minutes, followed by rotation at 10 rpm. Mice were allowed to walk on the bar for up to 300 seconds, and this was repeated 3 times. For the experimental measurements, the rotarod was programmed to ramped up from 10 rpm to 40 rpm over 300 seconds. Latency to fall was recorded over 3 trials with at least 15 minutes of rest between trials. All 3 measurements were averaged.

### Glucose and insulin measurements.

In the first cohort, glucose homeostasis was measured by evaluating blood glucose response during a glucose-tolerance test (GTT) at 8 and 19 weeks after doxycycline and by measuring blood glucose and insulin levels after an overnight fast and 4-hour refeeding period at 12 weeks after doxycycline. GTTs were performed on overnight-fasted mice, and blood glucose measurements were recorded using a ReliOn glucometer. A 20% glucose solution in saline was injected i.p. at 10 μL/gram of mouse weight to achieve a dose of 2 g/kg, and blood glucose was measured from tail blood at baseline and after 15, 30, 45, 60, and 120 minutes. Lactate measurements were recorded from tail blood using a LactatePLUS meter (Nova Biomedical). For the fast/refeed, blood glucose measurements were made using the glucometer and 20 μL of tail blood was harvested in an EDTA-coated microvette tube from a nonrestrained mouse for insulin measurements during the fasted state and again after 4 hours of refeeding. Blood samples were centrifuged at ~2,200 *g* for 5 minutes to separate plasma, and plasma samples were stored at –80°C. Plasma insulin was measured in duplicate by double-antibody radioimmunoassays (MilliporeSigma).

### Metabolite extraction and untargeted and targeted metabolomics.

Heart and cerebellum samples collected at sacrifice were freeze clamped in liquid nitrogen and stored at –80°C. Samples were then weighed on dry ice and ground to powder in 2 mL round-bottom tubes cooled in liquid nitrogen using a CryoMill homogenizer for 30 seconds at 25 Hz. For every 20 mg tissue, 800 μL –20°C 40:40:20 (v/v/v) acetonitrile/methanol/water solution was added to the tube. For 100 μg of mitochondrial pellet, 65 μL –20°C 40:40:20 (v/v/v) acetonitrile/methanol/water solution was added to the tube. Extracts were vortexed for 10 seconds and were then centrifuged at 21,000*g* for 20 minutes at 4°C. The supernatants were then transferred to plastic vials for liquid chromatography–mass spectrometry (LC-MS) analysis. A procedure blank was generated identically without tissue and was used later to remove the background ions.

For untargeted metabolomics, LC separation was achieved using a Vanquish UHPLC system (Thermo Fisher Scientific) with an Xbridge BEH Amide column (150 × 2 mm, 2.5 μm particle size; Waters). Solvent A is 95:5 water/acetonitrile with 20 mM ammonium acetate and 20 mM ammonium hydroxide at pH 9.4, and solvent B is acetonitrile. The gradient was 0 minutes, 90% B; 3 minutes, 75% B; 8 minutes, 70% B; 10 minutes, 50% B; 13 minutes, 25% B; 16 minutes, 0% B; and 21 minutes, 90% B. The total running time was 25 minutes at a flow rate of 150 μL min−1. LC-MS data were collected on a Q-Exactive Plus mass spectrometer (Thermo Fisher Scientific) operating in full scan mode with an MS1 scan range of *m/z* 70–1,000 and a resolving power of 140,000 at *m/z* 200. Other mass spectrometer parameters were as follows: sheath gas flow rate, 28 (arbitrary units); auxiliary gas flow rate, 10 (arbitrary units); sweep gas flow rate, 1 (arbitrary units); spray voltage, 3.5 kV; capillary temperature, 320°C; S-lens radiofrequency level, 65; automatic gain control (AGC) target, 3E6; and maximum injection time, 500 ms.

For targeted metabolomics, LC separation was achieved using a Vanquish UHPLC system (Thermo Fisher Scientific) with an Xbridge BEH Amide column (150 × 2 mm, 2.5 μm particle size; Waters). Solvent A was 95:5 water/acetonitrile with 20 mM ammonium acetate and 20 mM ammonium hydroxide at pH 9.4, and solvent B is acetonitrile. The gradient was 0 minutes, 85% B; 3 minutes, 60% B; 9.5 minutes, 35% B; 12.5 minutes, 0% B; and 18 minutes, 85% B. The total running time was 20 minutes at a flow rate of 150 μL min^–1^. LC-MS data were collected on a Q-Exactive Plus mass spectrometer (Thermo Fisher Scientific) operating in positive scan mode with an MS1 scan range of *m/z* 70–800 and a resolving power of 140,000 at *m/z* 200. Other mass spectrometer parameters were as follows: sheath gas flow rate, 28 (arbitrary units); auxiliary gas flow rate, 10 (arbitrary units); sweep gas flow rate, 1 (arbitrary units); spray voltage, 3.3 kV; capillary temperature, 320°C; S-lens radiofrequency level, 65; AGC target, 3E6; and maximum injection time, 500 ms.

LC-MS raw data files (.raw) were converted to mzXML format using ProteoWizard (version 3.0.20315). El-MAVEN (version 0.12.0) was used to generate a peak table containing *m/z*, retention time, and intensity for the peaks. Parameters for peak picking were the defaults except for the following: mass domain resolution, 5 ppm; time domain resolution, 10 scans; minimum intensity, 10,000 counts per second; and minimum peak width, 5 scans. The resulting peak table was exported as a .csv file. Peak annotation of untargeted metabolomics data was performed using NetID with default parameters. Individual metabolite abundance was normalized to total signal from all identified metabolites in a single sample.

### Transcriptomics.

RNA was prepared using Qiagen RNEasy kits. Briefly, 1–2 mg of heart tissue was homogenized by hand using a small pestle fitted to a 1.5 mL centrifuge tube in RLT Buffer. Once the homogenized sample was prepared, RNA was isolated according to the manufacturer’s instructions. RNA-Seq and analysis were performed by Novogene Inc. After mRNA purification and library preparation, quantification of gene expression levels was performed using featureCounts v1.5.0-p3, and the resulting data were used to calculate fragments per kilobase per million base pairs (FPKM). Differential expression analysis of 2 conditions/groups (2 biological replicates per condition) was performed using the DESeq2 R package (1.20.0). The resulting *P* values were adjusted using the Benjamini and Hochberg’s approach for controlling the FDR. Genes with an *P*_adj_ ≤ 0.05 found by DESeq2 were assigned as differentially expressed. Gene ontology (GO) enrichment analysis of differentially expressed genes was implemented by the clusterProfiler R package, in which gene length bias was corrected. GO terms with corrected *P* value less than 0.05 were considered significantly enriched among differentially expressed genes. Finally, gene set enrichment analysis (GSEA) was performed to test whether predefined gene sets were overrepresented among up- or downregulated genes.

### Quantitative PCR (qPCR).

RNA was prepared as above. cDNA was generated using the High Capacity cDNA Reverse Transcription Kit (Applied Biosciences) according to manufacturer’s instructions. Frataxin level was determined relative to actin using SYBR-Green relative qPCR (ddCT). *Fxn:* FWD 5′-GCTGGAGGGAACCGATCGTA-3′, REV 5′-TTCCTCAAATGCACCACGCAG; *Actb:* FWD 5′-GGCTGTATTCCCCTCCATCG-3′, REV 5′-CCAGTTGGTAACAATGCCATGT-3′.

### Mitochondrial isolation.

Mitochondria were isolated from heart tissue as previously described (75). Briefly, the heart was rapidly sectioned on ice and placed into ice-cold mitochondrial isolation buffer (MIB, containing 210 mM mannitol, 70 mM sucrose, 1 mM EDTA, 10 mM HEPES, final pH adjusted to 7.2 using KOH and freshly supplemented with 0.25% fatty acid–free BSA). The tissue was then minced and collected into a homogenization glass with 1.5 mL MIB+BSA. The tissue was homogenized using a Teflon Potter-Elvehjem pestle connected to a rotor for 10–12 strokes at 600 rpm. The homogenate was transferred to a microcentrifuge tube and centrifuged at 700 *g* for 10 minutes at 4°C. The supernatant containing mitochondria was then transferred to a new microcentrifuge tube, and the pellet was reconstituted in MIB+BSA and centrifuged for a second time at 700 *g* to retrieve additional mitochondria. The supernatants were combined into 1 tube and centrifuged at 11,800 *g* for 10 minutes at 4°C to pellet mitochondria. The mitochondrial pellet was then washed with 500 μL of MIB and centrifuged again at 11,800 *g* for 10 minutes. The final pellet was resuspended in 100–200 μL of MIB, and yield was determined by absorbance using a Nanodrop (Protein A280) and confirmed by BCA protein quantification.

### Mitochondrial respiration assays.

Respiration assays on isolated mitochondria were performed in an Oroboros O2k High Resolution Respirometer (Oroboros Instruments). For measurement of complex I, II, and IV respiration, 75–100 μg of heart mitochondria or 250 μg of liver mitochondria were diluted into the chamber containing 2.1 mL of MIRO5 (110 mM sucrose, 20 mM HEPES, 20 mM taurine, 60 mM K-lactobionate, 3 mM MgCl_2_, 10 mM KH_2_PO_4_, 0.5 mM EGTA, fatty acid free BSA 1 g/L [Fraction V, Roche], pH adjusted to 7.1 using KOH) respiration buffer supplemented with pyruvate (20 mM) and malate (10 mM). After the chamber was sealed, the signal was allowed to stabilize for 5–10 minutes. ADP (2.5mM) was added to initiate state 3 respiration. Pericidin (0.5 μM) was added to stop complex I respiration, and succinate (20 mM) was added to initiate complex II respiration. Antimycin A (2.5 μM) was then added to stop complex I– and II–dependent respiration. We then added N, N, N′, N′-tetramethyl-p-phenylenediamine (TMPD, 2 mM) along with ascorbic acid (0.5 mM), followed by sodium azide (5 mM) to measure complex IV respiration. Complex I (CI) and complex II (CII) were quantified by subtracting the average value following antimycin A addition from the rates obtained using pyruvate, malate, and ADP (CI) or succinate (CII), respectively. Complex IV was quantified by subtracting the respiration rate after azide addition from the TMPD/ascorbate value. Fatty acid oxidation was measured by adding malate (10 mM) followed by palmitoyl-carnitine (0.02 mM) and ADP (2.5 mM).

### Histology.

Cross sections of heart muscle were rinsed in PBS and preserved in 4% PFA; they were then rinsed 3× in PBS over a minimum of 2 days and transferred to 70% ethanol at least overnight (18–72 hours) and then 100% ethanol until prepared for sectioning. The tissue was sectioned and stained with H&E or Masson’s trichrome blue. Representative photomicrographs were taken at 40× magnification using a light microscope (Olympus DP72) coupled with a digital image acquisition system. Fibrosis was quantified from images at ×40 magnification. The total tissue and fibrotic tissue (blue stain) areas were quantified using ImageJ (NIH) adjusted color threshold functions and expressed as a percentage of the total areas that was fibrotic.

### Cardiac record study patient echocardiography data.

Human echocardiographic data were derived from participants in the Friedreich Ataxia Clinical Outcome Measure Study (FACOMS) (70) who also participated the CHOP-specific cardiac records study (6). These 2 long-running natural history studies have 406 joint participants who have contributed medical records containing 2,540 echoes. Nintey-one patients had either no EF, or all studies were already from later-stage cardiomyopathy with loss of systolic function (EF < 50%), leaving 315 patients for analysis. From these records, the echocardiograph with the highest EF for each patient was used. A subset of patients with data recordings that spanned at least 6 years (*n* = 106) was used to examine the pattern of EF changes over time. Data analyzed included EF and peak EF, and diastolic posterior wall thickness (PWTd) at the visit on which peak EF occurred. Interventricular septum (IVS) values were normalized for body surface area. Statistical calculations were performed in Stata and GraphPad. Reference values for healthy controls were obtained by using the mean and SD of reported EFs in adolescents to simulate a data set (*n* = 316) with Gaussian distribution (mean EF = 64.94 ± 3.25) (56). Similarly, reference values for diastolic PWTd at median age 24 were used to simulate a data set with gaussian distribution (mean PWTd = 8.62 cm ± 1.28 mm) (57).

### Statistics.

All results in this study are presented as mean ± SEM unless otherwise indicated. Two-tailed *t* tests were used to compare means between two groups. One-way ANOVA was used for comparing 3 or more groups followed by Fisher’s least-significant difference (LSD) test. Wald tests were used where indicated to test a hypothesis on a collective set of parameters between two groups. Statistical analysis was performed on GraphPad/Prism 9. *P* values less than 0.05 were deemed significant.

### Study approval.

All animal work was performed in accordance with the guidelines and with approval of the University of Pennsylvania IACUC and the CHOP IACUC. All clinical data were collected with the guidelines and approval of the CHOP IRB.

### Data availability.

All animal data supporting the findings of this study are available within the paper and its Supporting Data Values file. Deidentified patient data are provided to the extent possible and reasonable requests for additional data will be considered on a case-by-case basis. Data are located in controlled access data storage at CHOP. The GEO accession no. for RNA Seq data set is GSE271798 (76, 77). The Metabolights accession no. for Metabolomics is MTBLS10625 (78).

## Author contributions

The study was conceived and designed by CEP, SEM, DRL, and JAB. CEP completed experimental animal work including GTTs, rotarod evaluation, and echocardiography and performed animal and human data analysis. SMH and LVN prepared and maintained animal colonies at CHOP. SM and BC assisted animal sacrifice, tissue preparation, and respirometry. ML provided training and guidance for echocardiography measurements. JGD prepared and maintained animal colonies at University of Pennsylvania and performed survival study. GKA quantified fibrosis staining. WDL performed LC-MS on all samples under the supervision of JDR. APS and IH assisted with colony preparation, sacrifice, mitochondrial respiration, and NAD^+^ measurements. ANB and DDB assisted with echocardiography preparation, sacrifice, and mitochondrial isolation. EMA and LPF provided staining and analysis of cerebellum. SEM, JT, AD, SDS, DMB, KW, KYL, and DRL provided access to human data and reviewed the manuscript. DJL provided guidance and study materials. DRL analyzed and deidentified echocardiography data from FACOMS participants and provided animal housing. CEP and JAB prepared the manuscript, and all coauthors reviewed and provided feedback on the text.

## Supplementary Material

Supplemental data

Supplemental table 1

Supplemental table 2

Supplemental table 3

Supplemental table 4

Supplemental table 5

Supporting data values

## Version 1 08/22/2024

Electronic publication
