# Supplementary material for: NAD+ precursors prolong survival and improve cardiac phenotypes in a mouse model of Friedreich’s Ataxia
Source: JCI Insight. 2024 Jul 18;9(16):e177152. doi: 10.1172/jci.insight.177152 (PMC11343603; doi:10.1172/jci.insight.177152)
Supplement: Supplemental data [file jciinsight-9-177152-s263.pdf]

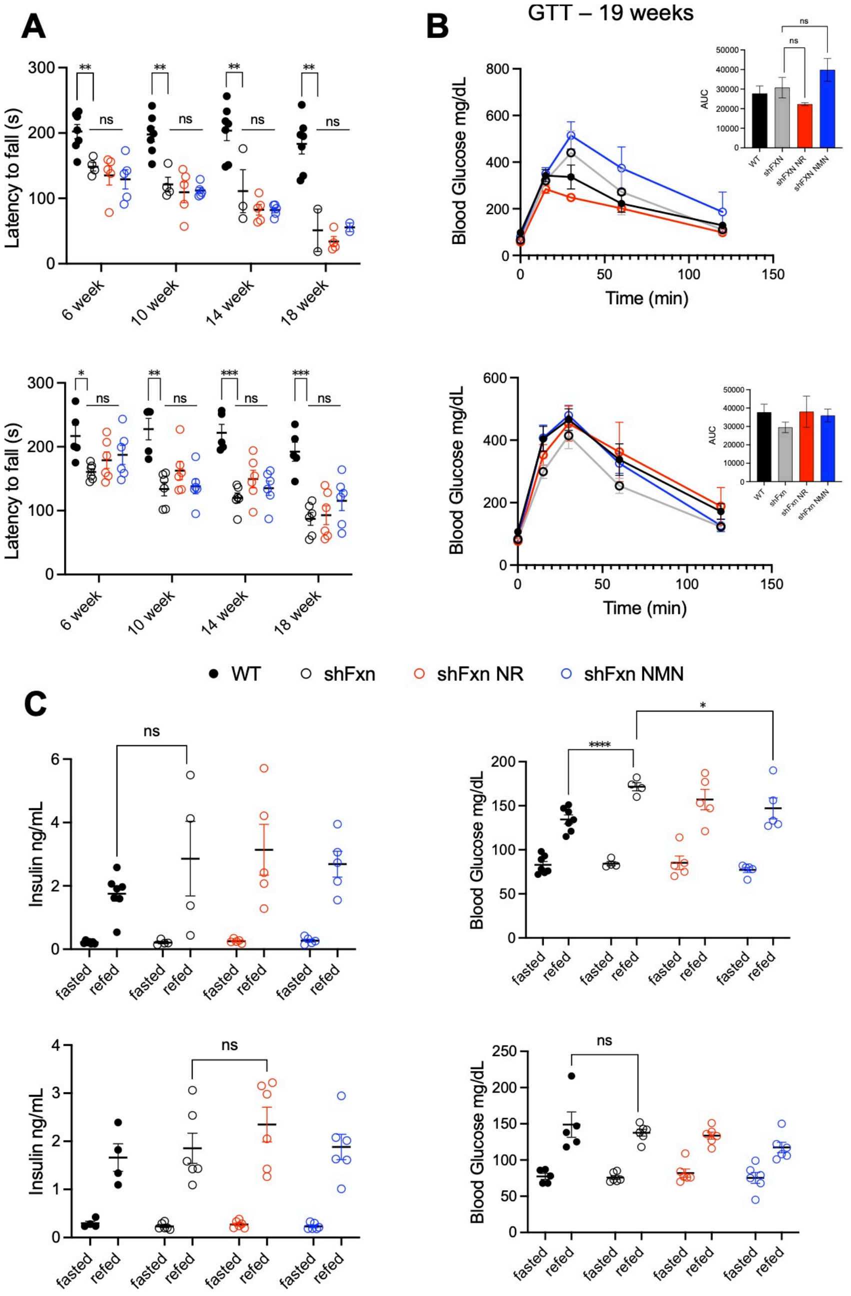


**Figure S1. Treatment with NAD$^+$ precursors does not consistently affect rotarod performance or glucose homeostasis in shFXN mice.** (a) Latency to fall on rotarod in males (top) and females (bottom) (n=2-17) (b) Glucose tolerance test at 19 weeks post-doxycycline (n=3-7) (b, insets) Area under curve for respective GTT (c) Fasting and 4-hour refed insulin and blood glucose levels (n=4-7). (a, c: Two-Way ANOVA) (b): One-Way ANOVA) (*=p≤.05, **=p≤0.01, ***=p≤0.001, ****=p≤0.0001)

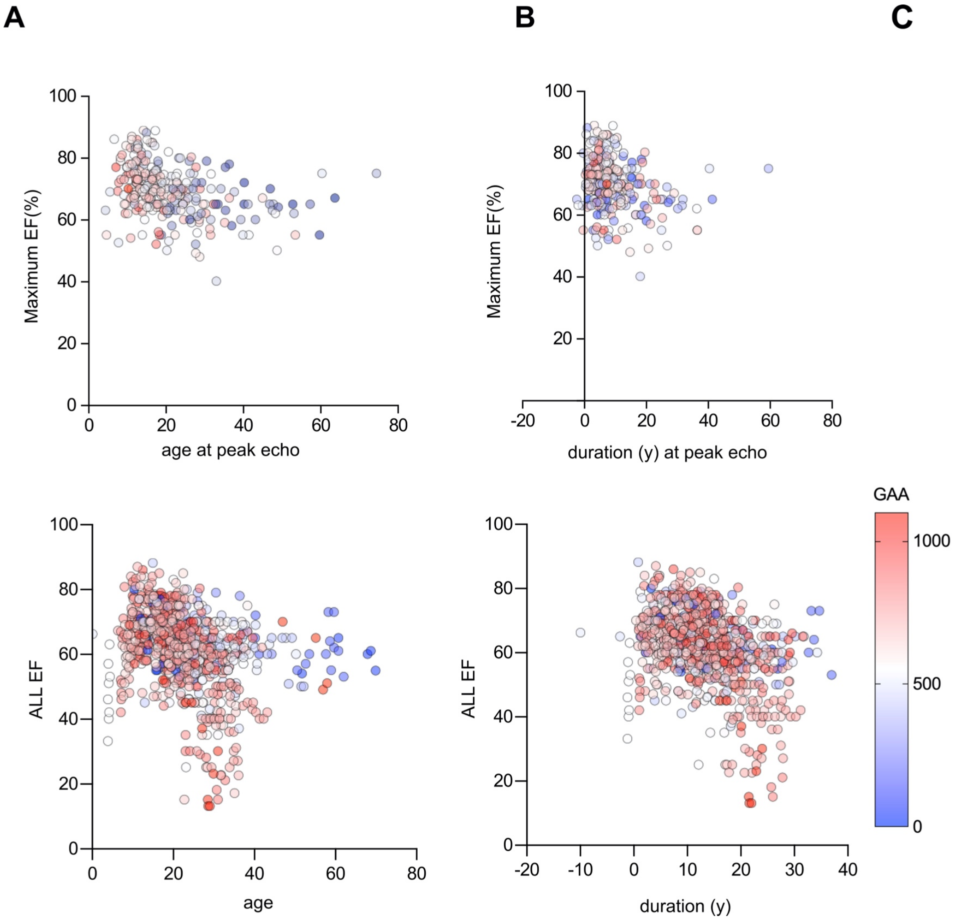


C

| Characteristics | FRDA individuals with Maximal Echo record (N=323) |
|---|---|
| Age of onset | 11.7±7.1 |
| Male sex | 157 (48.6) |
| GAA repeat length | 674±217 |
| Age at record | 20.3±11.2 |

| Heart Medication Usage | Individuals with FRDA (N=338) |
|---|---|
| ALL | 127 (37.6) |
| ACE Inhibitors | 54 (15.9) |
| Angiotensin II Inhibitors | 4 (1.2) |
| Anticoagulants | 36 (10.7) |
| Beta blockers | 88 (26) |
| $Ca^{2+}$-channel blockers | 14 (4.2) |
| Cholesterol-lowering medications | 17 (5.0) |
| Diuretics | 11 (3.3) |
| Other | 15 (4.4) |

**Figure S2. High EFs are common in adolescents with FRDA while lower EFs are generally seen between 20 and 40 years of age.** (a) Maximal EF (top) and all EFs recorded over 6+ years (bottom) according to age at echocardiogram (n=330 top, n=106 bottom) (b) Maximal EF (top) and all EFs recorded over 6+ years (bottom) according to disease duration (time since diagnosis) at echocardiogram (n=330 top, 106 bottom). (c) Clinical characteristics of FRDA dataset (top) and heart medication usage tabulation (bottom) in large cohort (n=338)

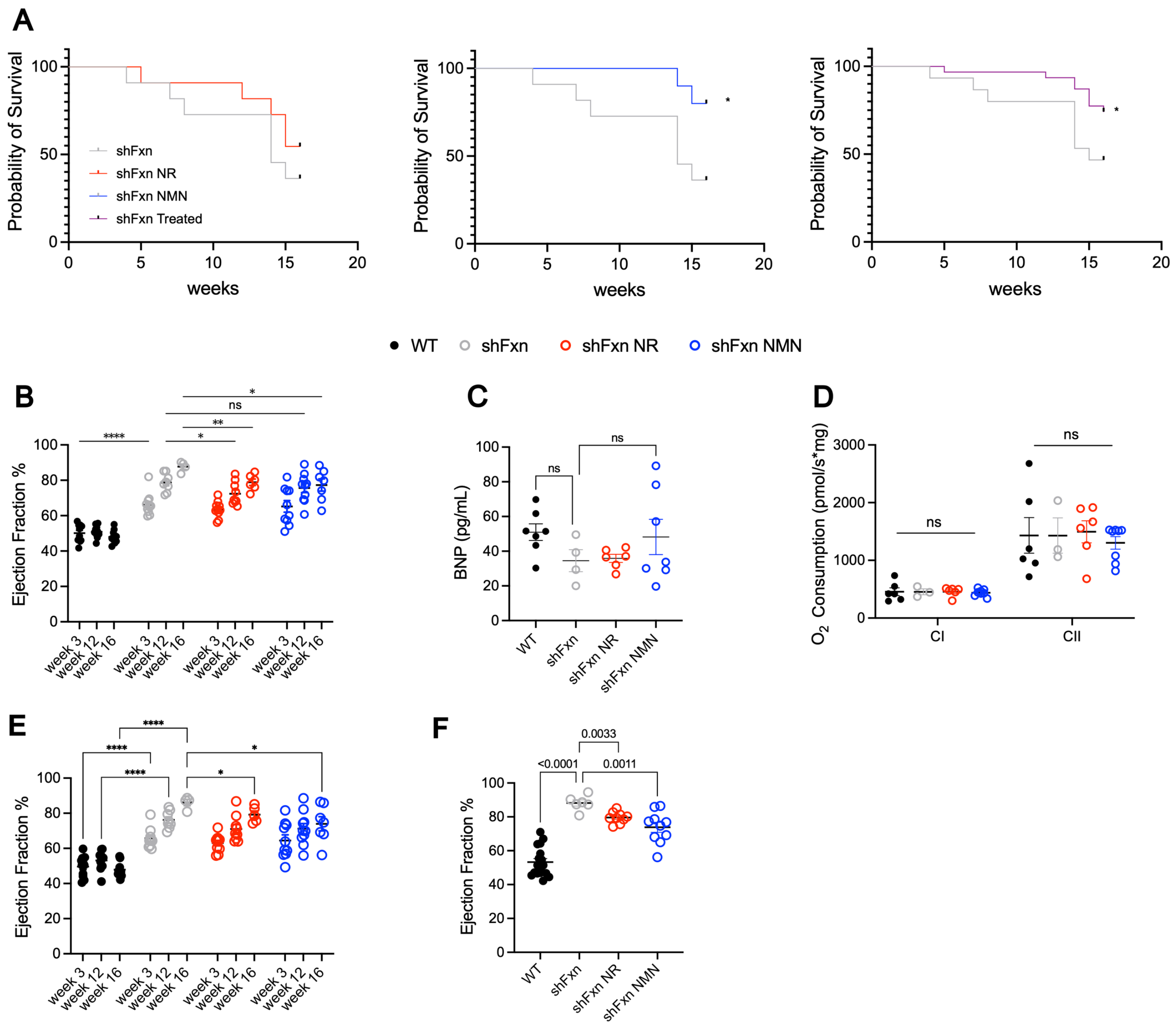


**Figure S3. Analyses restricted to the 16-week cohort and short axis only measurements** (a) Male shFxn survival curves with treatment (left, center: n=10-11, cohort 2 only, right: n=15-31 all treated) (b) Ejection fraction at 3, 12, and 16 weeks post-doxycycline (n=4-10) (c) BNP Levels in plasma at 16 weeks post-doxycycline (n=4-8) (d) Liver mitochondrial complex I and complex II respiration (n=3-8) (e) longitudinal measurement of ejection fraction calculated with short axis measurement only (f) Ejection fraction at 16-18 weeks calculated with short axis only. (a: Pairwise Mantel-Cox test for Survival) (b,d,e: Two-Way ANOVA) (c,f: One-Way ANOVA). (*=p≤.05, **=p≤0.01, ***=p≤0.001, ****=p≤0.0001)

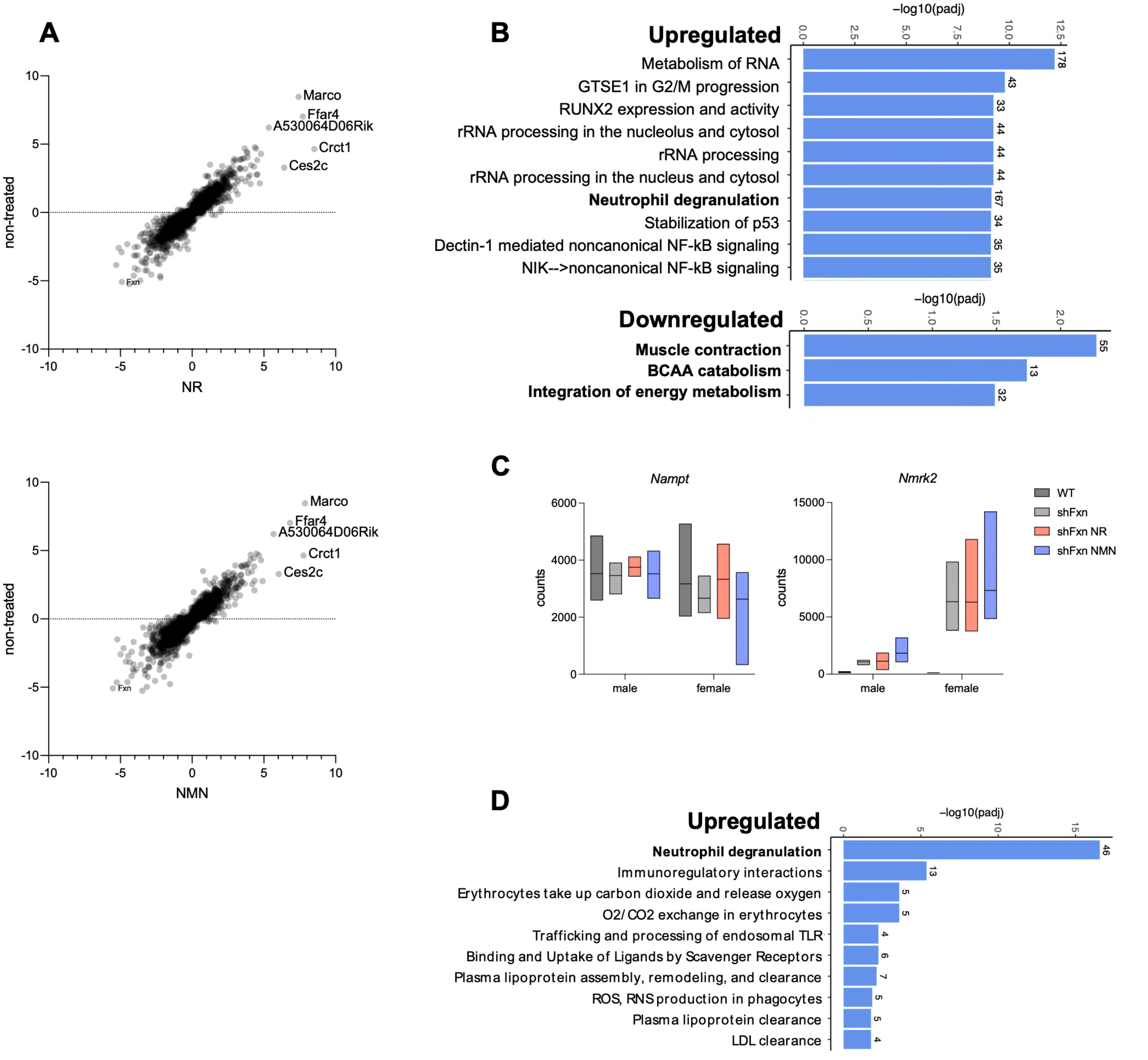


**Figure S4. Transcriptomic signatures in shFxn mice.** (a) Non-treated versus NR (top) and NMN (bottom) $\log_2$(FC) relative to WT. (b) Upregulated and downregulated reactome terms in female shFxn mice (shFxn vs WT) (c) *Nmrk2* and *Nampt* levels in male and female mice (d) Upregulated terms in 20-week (experiment 1) versus 16-week (experiment 2) shFxn male mice.

**A**

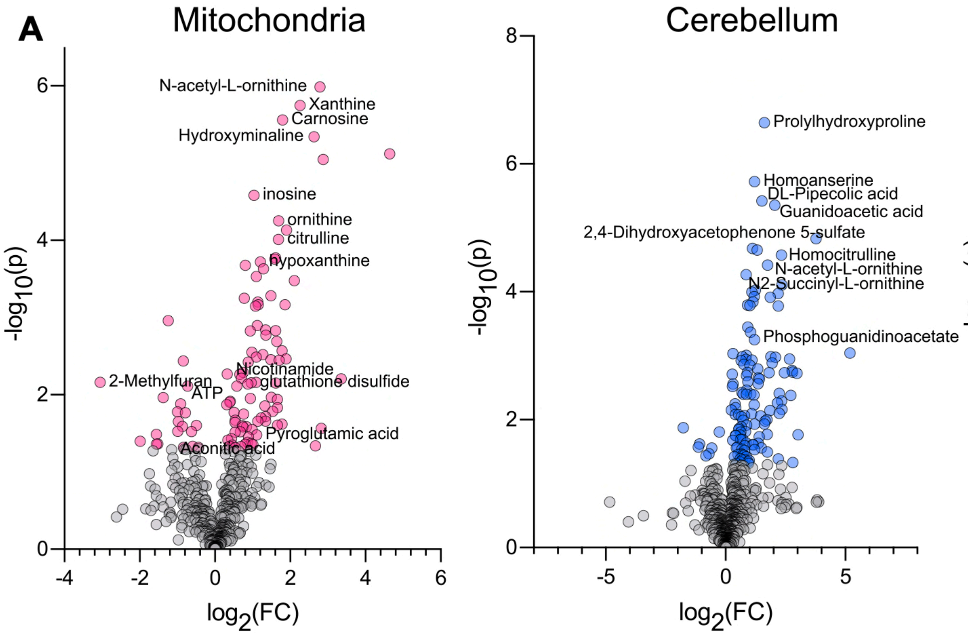


**B**

| | Metabolite | $log_2$(FC) | p |
|---|---|---|---|
| TCA | **pyruvate** | -0.20 | ns |
| | **citrate/isocitrate** | 0.10 | ns |
| | **alpha-ketoglutarate** | -0.14 | ns |
| | **succinate** | -0.57 | 2.0E-04 |
| | **fumarate** | -0.67 | 9.2E-05 |
| | **malate** | -0.67 | 5.4E-05 |
| | **aspartate** | -0.64 | ns |
| BCAA | **leucine/isoleucine** | -0.41 | 3.3E-02 |
| | **valine** | -0.45 | 9.1E-03 |
| | **2 methyl 3 ketovaleric acid** | -1.15 | 1.4E-02 |

**Figure S5. Metabolomic profiles of heart mitochondria, cerebellum, and plasma** (a) Volcano plot of mitochondria, cerebellum, and plasma (b) TCA and BCAA metabolites in heart tissue. (n=4-10, 2-tailed ttest, nominal p)

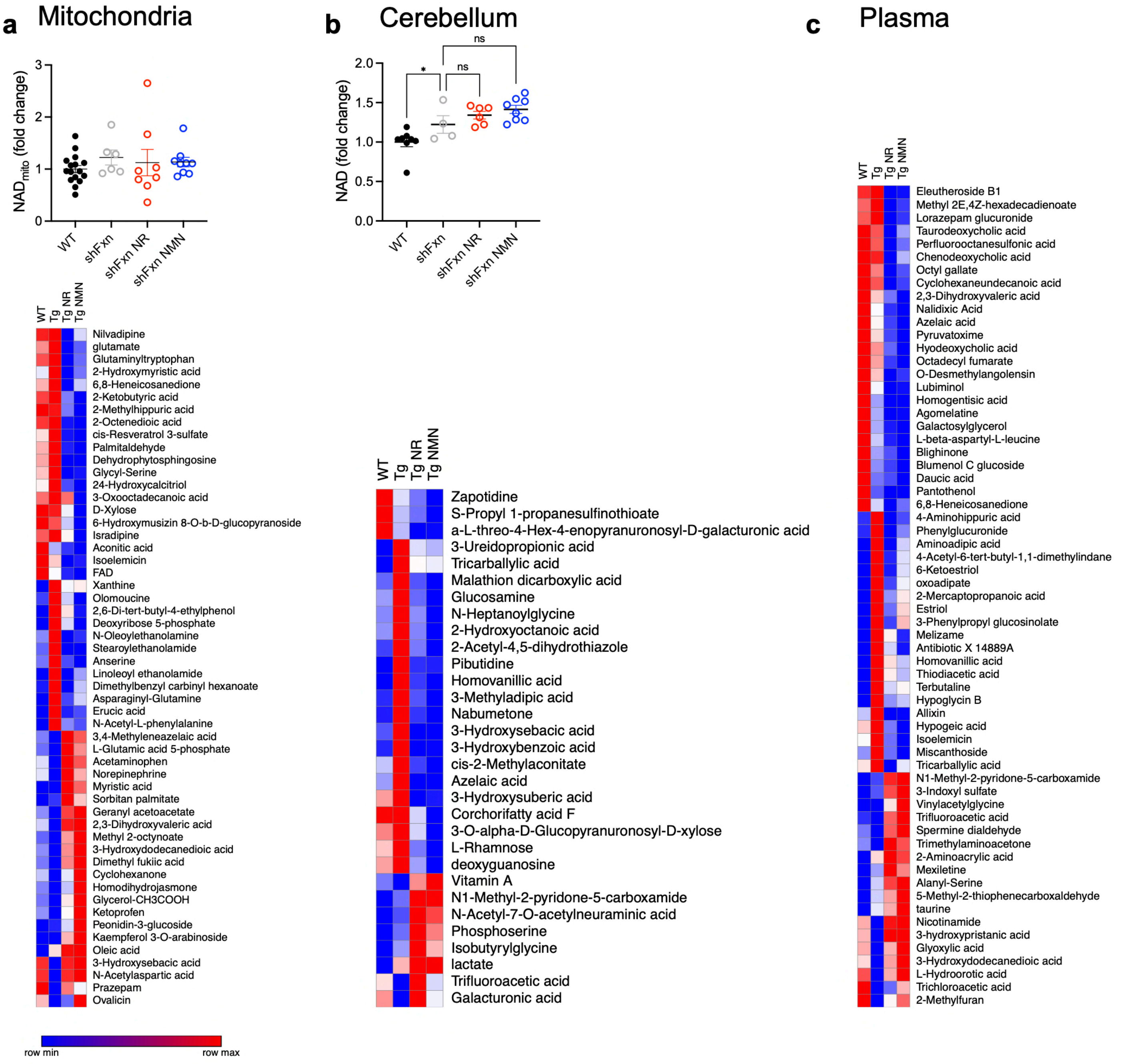


**Figure S6. Metabolic effects of NAD precursor treatments in mitochondria, cerebellum, and plasma.** (a) Mitochondrial $NAD^+$ 16-20 weeks post-doxycycline in males (b) Cerebellum $NAD^+$ 16 weeks post-doxycycline in males (c) NR and NMN changes in mitochondria, cerebellum, and plasma (n=4-10,normalized MS value, 2-tailed ttest nominal p) (*=p≤.05, **=p≤0.01, ***=p≤0.001, ****=p≤0.0001)

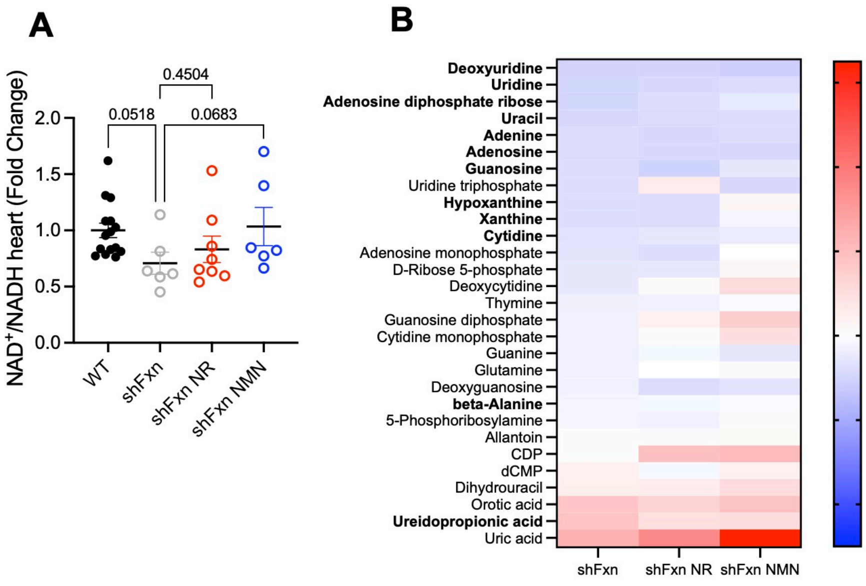


**C**

| *Nrf2* target | $log_2(FC)$ | padj |
|---|---|---|
| ***Gclm*** | 0.88 | 5.5E-08 |
| ***Gclc*** | 0.64 | 8.9E-10 |
| ***Slc7a11*** | 3.76 | 5.1E-05 |
| ***Mthfd2*** | 0.81 | 2.1E-02 |
| ***Blvra*** | 0.47 | 1.5E-03 |
| ***Blvrb*** | 0.92 | 2.7E-07 |
| ***Hmox1*** | 3.94 | 2.2E-109 |
| ***Ftl1*** | 1.33 | 1.0E-18 |
| ***Gbe1*** | 0.32 | 1.8E-02 |
| ***Phgdh*** | 0.86 | 3.8E-02 |
| ***Hk3*** | 2.04 | 1.0E-07 |
| ***Srxn1*** | 1.12 | 1.7E-11 |
| ***Gsr*** | 0.76 | 2.1E-08 |
| ***Aldob*** | -1.04 | 1.9E-03 |
| ***Pkm*** | -0.33 | 4.6E-04 |
| ***Pfkl*** | -1.29 | 3.2E-47 |
| ***Gpi1*** | -0.28 | 9.8E-03 |
| ***Hk1*** | -0.34 | 2.0E-02 |
| ***Hk2*** | -0.76 | 5.0E-09 |
| ***Nqo1*** | -0.5 | 1.0E-03 |
| ***Pgm5*** | -0.48 | 2.6E-03 |

**Figure S7. Nrf2 targets are modified in shFxn mouse hearts.** (a) $NAD^+/NADH$ ratio in male shFxn hearts (n=6-15, One-Way ANOVA) (b) de novo nucleotide synthesis metabolites (bolded are significant in WT vs shFxn, n=4-8, Unpaired 2-tailed t-test) (c) Upregulated (red) and downregulated (blue) Nrf2 targets by counts (n=4-5, Wald Test)
